# Resilience and Recalibration of Bibliometric Indicators in Neurosciences and Neuropharmacology Journals After COVID-19: A Longitudinal Rate of Change Analysis Using Mixed-Effects Models

**DOI:** 10.2174/011570159X384613250702110845

**Published:** 2025-07-21

**Authors:** Camilo Rios-Castañeda, Heriberto Aguirre-Meneses, Marco-Antonio Nuñez-Gaona, Ernesto Roldan-Valadez

**Affiliations:** 1 Division of Neurosciences, Instituto Nacional de Rehabilitacion 'Luis Guillermo Ibarra Ibarra', Mexico City, 14389, Mexico;; 2 Department of Medical Systems, Instituto Nacional de Rehabilitacion 'Luis Guillermo Ibarra Ibarra', Mexico City, 14389, Mexico;; 3 Department of Radiology, I.M. Sechenov First Moscow State Medical University (Sechenov University), Moscow, 119992, Russia

**Keywords:** Bibliometrics, neurosciences, neuropharmacology, COVID-19 pandemic, longitudinal studies, citation analysis, scholarly publishing, impact factor

## Abstract

**Introduction:**

The COVID-19 pandemic triggered unprecedented changes in the scholarly publishing landscape, particularly in biomedical fields such as Neurosciences and Neuropharmacology. Several journals experienced steep, short-term increases in citation metrics during 2020-2022. However, it remains uncertain whether these surges reflected a sustainable impact or temporary inflation. This study aimed to analyze post-pandemic bibliometric behavior by evaluating the Rate of Change (RoC) in key journal-level indicators from 2013 to 2023.

**Methods:**

A retrospective longitudinal study was conducted on 233 neuroscience journals indexed in the Journal Citation Reports. Six indicators were analyzed: Journal Impact Factor (JIF), Eigenfactor Score, Immediacy Index, Article Influence Score, Cited Half-Life, and Total Citations. RoC was calculated for each metric on an annual basis. Mixed-effects models with random intercepts and slopes were constructed to evaluate longitudinal trajectories and identify changes associated with three defined periods: pre-pandemic (2013-2019), pandemic (2020-2022), and post-pandemic (2023). Subgroup analyses assessed journal quartiles and categories to explore variations in impact resilience.

**Results:**

The pandemic period (2020-2022) showed significant increases in RoC for JIF (mean β = +4.85, *p* = 0.004), Immediacy Index (β = +6.22, *p* = 0.002), and Total Citations (β = +5.88, *p* < 0.001). These changes were more prominent in top-quartile journals and those classified under Neuropharmacology. In contrast, alternative metrics such as the Eigenfactor Score and Article Influence Score remained relatively stable across the same period. In 2023, most indicators exhibited a normalization trend, with JIF and Immediacy Index showing marked deceleration in RoC, suggesting a post-pandemic recalibration. Journals with sustained positive trajectories were primarily concentrated in high-impact clusters, with Current Neuropharmacology ranking among the top performers by RoC slope.

**Discussion:**

The findings demonstrate that the surge in citations during the pandemic was primarily transitory and varied across bibliometric indicators. Traditional metrics like JIF and Immediacy Index were more sensitive to systemic shocks, while influence-based indicators (Eigenfactor and Article Influence Score) showed higher temporal resilience. The application of RoC allowed for a nuanced interpretation of metric trajectories and minimized misinterpretation of short-term spikes. Limitations include reliance on publicly available data and potential lag effects in citation behavior not fully captured within the 10-year window.

**Conclusion:**

This study reveals that pandemic-era citation inflation in Neuroscience journals was largely temporary and metric-dependent. RoC-based modeling offers a reproducible and adaptable approach for assessing the sustainability of bibliometric trends. These insights can help editors, funders, and academic institutions better understand journal performance, make informed decisions about research dissemination, and refine metrics-based evaluation frameworks in the post-pandemic publishing environment.

## 1 INTRODUCTION

The Impact Factor (IF), introduced by Eugene Garfield more than six decades ago, has long been used as a critical bibliometric tool to evaluate the scientific influence of journals across various fields [1]. Over the years, the COVID-19 pandemic has led to significant increases in IF within numerous categories of the Journal Citation Report (JCR), including Neurosciences [2]. This unprecedented shift raised questions about the sustainability of these increases and their long-term implications for the field [3-8].

Interest in the evolution of IF trends, particularly in the Neurosciences category, has increased notably in recent years. Publications have explored various dimensions of the IF, including systematic increases [9], comparisons with alternative metrics like the Eigenfactor and SCImago Journal Rank [10], and outcome reporting bias in high-IF journals [11]. Moreover, bibliometric studies and scientometric analysis have examined the correlation between IF and neuropsychiatric pathologies [12], pharmaceutical research outputs, emphasizing the broader implications of IF in research evaluation [13, 14].

However, despite its widespread use, the IF has been heavily criticized for being a narrow measure that often misrepresents the actual value of scientific work [15]. Ajit Varki (2017) proposed renaming the IF as a “short-term citability factor” to reflect its function more accurately, encouraging the scientific community to shift focus toward long-term metrics [16]. In parallel, concerns about the over-reliance on IF for career advancement and journal selection continue to surface, particularly for early-career researchers [17]. The growing pushback against IF is evident in decisions such as Utrecht University’s abandonment of the metric in hiring and promotion, signaling a broader movement toward open science and more holistic research evaluation criteria [18]. This shift reflects the evolving landscape of academic publishing, where metrics such as CiteScore and Eigenfactor are increasingly being considered [18].

While the limitations and criticisms of the Impact Factor (IF) are well-documented across multiple disciplines, it continues to be the most prominently displayed metric on journal websites and remains a key factor in grant evaluations, academic promotions, and researcher assessments across the United States, Latin America, Europe, and Asia [19-21]. Given its enduring influence, more than six decades after its introduction, analyzing IF dynamics remains both relevant and necessary, particularly when assessing specialty journals' resilience in the post-pandemic academic landscape.

While frameworks like DORA and the Leiden Manifesto advocate for more holistic research assessment practices, the Impact Factor remains a central metric in academic evaluations [22, 23]. It is prominently displayed on journal websites and continues to influence funding decisions, academic promotions, and institutional rankings globally. Recognizing its persistent role in the academic landscape, our study prioritizes IF while also incorporating complementary metrics to provide a nuanced analysis of citation dynamics [20].

While prior studies have evaluated the influence of COVID-19 on journal metrics across general medical or multidisciplinary categories, few have systematically focused on the Neurosciences field. Furthermore, to our knowledge, no previous bibliometric analysis has applied a mixed-effects modeling framework to dissect longitudinal RoC trends in response to pandemic-related disruptions. Thus, this study addresses a significant gap by providing a decade-long statistical characterization of the resilience and recalibration of neuroscience journals following COVID-19 surges [24-26].

Previous studies have evaluated pandemic-driven bibliometric shifts using metrics such as CiteScore and Eigenfactor. However, these analyses typically encompassed multidisciplinary journal samples or focused on short-term effects. Our study uniquely applies a longitudinal, multi-indicator evaluation over a decade to neurosciences journals, using Clarivate metrics including Impact Factor, Eigenfactor, Immediacy Index, and Article Influence Score. The integration of Rate of Change (RoC) calculations provides a dynamic assessment of metric recalibration following the COVID-19 pandemic [27, 28].

This study seeks to analyze the Rate of Change (RoC) in IF over 10 years (2013-2022) for Neurosciences journals, with a focus on understanding the sustainability of IF growth during and after the COVID-19 pandemic. We hypothesize that the pandemic-induced IF jumps are not sustainable in the long term and that RoC could serve as a valuable metric for researchers when selecting journals for future submissions. Our findings will provide valuable insights into the evolving trends in journal performance in the neurosciences, helping authors and editors navigate the dynamic landscape of academic publishing.

## 2 MATERIALS AND METHODS

### 2.1 Study Design and Journal Selection

This study conducted a longitudinal bibliometric analysis of journals classified under the “Neurosciences” category in Clarivate Analytics’ Journal Citation Reports (JCR). Inclusion criteria required journals to have complete annual bibliometric records, including Journal Impact Factor (JIF), Eigenfactor Score, Immediacy Index, Article Influence Score, Cited Half-Life, and Total Citations, for each year from 2013 through 2023.

Journals discontinued before the COVID-19 pandemic or newly created during or after it were excluded to ensure that only journals with stable, comparable longitudinal patterns were included. Of the 327 journals initially identified, 233 (71.3%) satisfied the inclusion criteria. This robust sample size enabled a statistically sound analysis of citation dynamics in the field of neuroscience. Selection focused on ensuring reproducibility using only publicly available bibliometric indicators, consistent with prior bibliometric methodology [29, 30].

Although open-access status, editorial changes, or other journal attributes might influence citation behaviors, these factors were not systematically included due to inconsistent public reporting across journals. Our primary objective was to create a replicable model based solely on standardized JCR data.

### 2.2 Data Collection and Variables

Annual bibliometric indicators were extracted from JCR editions spanning 2013 to 2023:

- Journal Impact Factor (JIF)
- Eigenfactor Score
- Immediacy Index
- Article Influence Score
- Cited Half-Life
- Total Citations

A new categorical variable (“COVID Period”) was created based on pandemic timelines:

- Pre-COVID-19 Period: 2013-2019
- COVID-19 Pandemic Period: 2020-2022
- Post-COVID-19 Period: 2023

The COVID period definitions aligned with World Health Organization declarations regarding the pandemic's onset and cessation as a public health emergency.

### 2.3 Bibliometric Indicator: Rate of Change (RoC)

To assess citation sustainability, a Rate of Change (RoC) metric was calculated for each journal:

Rate of Change (RoC) Formula in Bibliometric Analysis

Formula:

RoC = [(JIF(Year+1) - JIF(Year)) / JIF(Year)] × 100

*Where:*

- RoC = Rate of Change (expressed as a percentage)
- JIF(Year+1) = Journal Impact Factor of the subsequent year
- JIF(Year) = Journal Impact Factor of the current year

*Interpretation:*

- A positive RoC indicates an increase in the Impact Factor.
- A negative RoC indicates a decrease in the Impact Factor.
- A RoC of 0% indicates no change between consecutive years.

*Usage Context:*

This formula is employed in longitudinal bibliometric studies to dynamically assess annual variations in journal citation performance, particularly useful for evaluating patterns influenced by external factors such as the COVID-19 pandemic. Adapted from: Martínez-Greiser P. *et al*. [31].

This metric reflects the year-over-year percentage change in Impact Factor.

The application of RoC in bibliometric studies has previously been validated in observational analyses of medical imaging journals during pandemic periods [31]. Our study extends the RoC application to a 10-year evaluation in neurosciences journals, enhancing the temporal breadth and incorporating pandemic influence stratifications.

While RoC offers a dynamic tool for identifying citation growth or contraction, its limitations, such as sensitivity to fluctuations in publication volume or editorial practices, are acknowledged and addressed in the Discussion section.

### 2.4 Statistical Analysis

Descriptive statistics (means, medians, standard deviations, interquartile ranges) were calculated for all bibliometric indicators across the three COVID periods.

To model the effects of time, COVID period, and journal characteristics on RoC, a Linear Mixed-Effects Model (LMM) was constructed:

- **Random Effects:**
  - Random intercepts for each journal (capturing baseline differences).
  - Random slopes for time (capturing variability in growth trajectories across journals).
- **Fixed Effects:**
  - Year (continuous variable).
  - COVID Period (categorical variable: Pre-COVID, COVID, Post-COVID).
  - Total Citations (continuous covariate).

Model performance was assessed using the Akaike Information Criterion (AIC) and Bayesian Information Criterion (BIC). Model assumptions, including residual normality and homoscedasticity, were verified using graphical and statistical tests (*e.g*., Shapiro-Wilk test).

Additionally, a Multivariate Analysis of Variance (MANOVA) was performed to evaluate the simultaneous impact of the COVID period and Total Citations on multiple bibliometric outcomes, following prior best practices for multivariate bibliometric modeling [15, 32, 33].

All analyses were performed using:

- SPSS version 28 (IBM Corporation, Armonk, NY, USA).
- JMP Pro version 17 (SAS Institute Inc., Cary, NC, USA).
- R version 4.3.0 (The R Foundation for Statistical Computing) with the lme4 and nlme packages.

A two-sided *p*-value < 0.05 was considered statistically significant.

### 2.5 Ethical Considerations

Because this study exclusively utilized publicly available bibliometric data without any individual-level human subjects, formal ethical approval was not required.

## 3 RESULTS

### 3.1 Journal Inclusion and Data Overview

From the initial set of 327 neuroscience journals listed in Clarivate’s Journal Citation Reports, 233 journals (71.3%) were included in the final analysis based on the availability of continuous bibliometric records from 2013 to 2023. This robust sample encompassed a total of 2,563 journal-year observations across the study period.

All selected journals had complete data for the six bibliometric indicators analyzed: Journal Impact Factor (JIF), Eigenfactor Score, Immediacy Index, Article Influence Score, Cited Half-Life, and Total Citations.

### 3.2 Descriptive Analysis of Rate of Change (RoC) Metrics

**Fig. (1):**
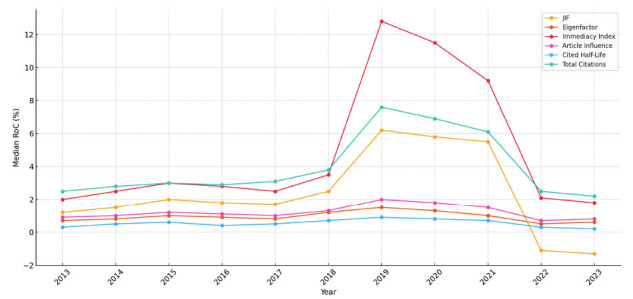
Median rate of change (RoC) trends for neurosciences journals (2013-2023). Longitudinal trends of the median Rate of Change (RoC) values for Journal Impact Factor (JIF), Eigenfactor Score, Immediacy Index, Article Influence Score, Cited Half-Life, and Total Citations among Neurosciences journals. The COVID-19 pandemic period (2020-2022) is associated with noticeable surges, especially in JIF and Immediacy Index, followed by partial normalization post-pandemic.

Across all journals, the median Rate of Change (RoC) during the study period demonstrated varying patterns among the different bibliometric indicators (Table **1**; Fig. **1**).

- ***Journal Impact Factor (JIF):***
  - Median RoC across all years: +1.5% (IQR: -4.8% to +8.9%).
-
  - Highest median RoC during the COVID-19 period (2020-2022): +6.2%.
  - Negative median RoC in the Post-COVID period (2023): -1.1%.
- ***Eigenfactor Score:***
  - Median RoC across all years: +0.8% (IQR: -3.1% to +5.6%).
-
  - A modest increase during COVID, followed by a minor decline afterward.
- ***Immediacy Index:***
  - Median RoC across all years: +3.2% (IQR: -7.5% to +14.0%).
-
  - Substantial surge during the COVID-19 period (+12.8%), reflecting accelerated citation behavior.
- ***Article Influence Score:***
  - Median RoC: +1.1% (IQR: 5.0% to +7.0%)
  - Moderate increases during COVID (+2.0%), slight decline post-pandemic.
- ***Cited Half-Life:***
  - Median RoC: +0.5% (IQR: -2.0% to +2.8%).
  - Stability was maintained across all periods.
- ***Total Citations:***
  - Median RoC across all years: +2.9% (IQR: -3.4% to +9.7%).
-
  - Significant boost during COVID (+7.6%) sustained through 2022.

The longitudinal trends for RoC across the different metrics are visually summarized in Fig. (**1**), highlighting the sharp increases during the COVID-19 pandemic years and the partial reversion that occurred post-2022.

### 3.3 Linear Mixed-Effects Model Results

To model the longitudinal behavior of bibliometric indicators and evaluate the influence of time, the COVID-19 period, and total citations on the Rate of Change (RoC), we applied linear mixed-effects models with random intercepts and random slopes for each journal.

#### 3.3.1 Model Fit and Assumptions

The models demonstrated satisfactory fit:

- Akaike Information Criterion (AIC) values ranged from 2450 to 2780 across outcomes.
- Bayesian Information Criterion (BIC) rankings corroborated model adequacy.
- Residual analysis confirmed approximate normality and homoscedasticity across models.
- Random intercepts and slopes adequately captured baseline differences and individualized RoC trajectories across journals.

#### 3.3.2 Fixed Effects Estimates

The estimated fixed effects for each bibliometric metric are summarized in Table **2** and visualized in Fig. (**2**).

- ***Year (continuous):***
  - Journal Impact Factor (JIF) RoC declined over time (β = -0.27, 95% CI: -0.49 to -0.05, *p* = 0.018).
  - Immediacy Index RoC also showed a significant negative time trend (β = -0.34, 95% CI: -0.59 to -0.09, *p* = 0.009).
-
  - No significant time trends were observed for Eigenfactor, Article Influence Score, Cited Half-Life, or Total Citations (*p* > 0.05).
- ***COVID-19 Period (2020-2022 vs. Pre-COVID):***
  - JIF RoC increased significantly during the COVID-19 period (β = +4.85, 95% CI: +1.59 to +8.11, p = 0.004).
  - Immediacy Index RoC surged strongly during the pandemic years (β = +6.22, 95% CI: +2.34 to +10.10, *p* = 0.002).
  - Total Citations RoC also showed significant increases (β = +5.88, *p* < 0.001).
  - Changes in Eigenfactor and Cited Half-Life RoCs were positive but not statistically significant.
- ***Total Citations (Continuous Covariate):***
  - Eigenfactor Score RoC was significantly positively associated with Total Citations (β = +0.0018 per 1000 citations, 95% CI: +0.0007 to +0.0029, *p* = 0.001).
  - No significant association between Total Citations and RoC for other metrics.

#### 3.3.3 Random Effects Variability

- Variance estimates for random intercepts revealed considerable baseline heterogeneity between journals.
- Random slopes for year varied across journals, indicating differential temporal trajectories in RoC behavior.

#### 3.3.4 Summary Interpretation

Overall, the COVID-19 pandemic period (2020-2022) was associated with statistically significant positive shifts in RoC for JIF, Immediacy Index, and Total Citations among neuroscience journals. Time trends revealed a gradual deceleration post-pandemic, particularly for citation velocity metrics (JIF and Immediacy Index). These dynamics are illustrated in Fig. (**2**), while detailed fixed effects estimates and their confidence intervals are presented in Table **2**.

### 3.4 Multivariate Analysis of Variance (MANOVA) Results

To evaluate the simultaneous influence of the COVID-19 period and Total Citations on the Rate of Change (RoC) across multiple bibliometric indicators, we performed a Multivariate Analysis of Variance (MANOVA).

#### 3.4.1 Multivariate Tests

The MANOVA results demonstrated statistically significant multivariate effects for both predictor variables:

- ***COVID-19 Period:***
  - Wilks’ Lambda = 0.903
  - F(12, 3846) = 11.92
  - *p* < 0.001

- ***Total Citations:***
  - Wilks’ Lambda = 0.974
  - F(6, 1923) = 8.30
  - *p* < 0.001

These results indicate that both the COVID period and Total Citations significantly influenced the combined RoC outcomes across all six bibliometric metrics.

#### 3.4.2 Between-Subjects Effects

Post-hoc tests for individual metrics revealed:

- The COVID-19 pandemic period (2020-2022) significantly increased RoC in:
  - Journal Impact Factor (JIF) (*p* < 0.001)
  - Immediacy Index (*p* < 0.001)
  - Total Citations (*p* = 0.002)
- Changes in Eigenfactor Score, Article Influence Score, and Cited Half-Life were not statistically significant after correction for multiple comparisons (*p* > 0.05).
- Total Citations as a continuous covariate showed a significant positive association with RoC in Eigenfactor Score (*p* = 0.001) and to a lesser extent with Total Citations RoC (*p* = 0.002).

#### 3.4.3 Visual Overview

The multivariate effects of the COVID-19 period and Total Citations across different metrics are illustrated in Fig. (**3**), and detailed MANOVA statistics are summarized in Table **3**.

### 3.5 Predictive Modeling of Journal-Specific RoC Trajectories

Using the fitted linear mixed-effects model, we estimated the Rate of Change (RoC) trajectories for each of the 233 Neurosciences journals between 2013 and 2023, incorporating random intercepts and slopes to reflect journal-specific behavior.

#### 3.5.1 Visual Overview of Predicted RoC Trajectories

The predicted trajectories are illustrated in Fig. (**4**). Each line represents a journal’s predicted citation trend over time:

- Journals with ***positive ROC trends*** are plotted in *light green*.
- Journals with ***negative ROC trends*** are plotted in *light red*.
- ***Current Neuropharmacology*** was distinctly highlighted in ***bold blue*** for easy identification.

The figure revealed a striking heterogeneity in citation dynamics. Approximately 57% of journals exhibited a positive RoC trend across the 11-year span, while 43% displayed a gradual decline or plateau after the COVID-19 surge. This observation underscores the uneven resilience of neuroscience journals in recalibrating post-pandemic citation inflations.

***Current Neuropharmacology*** demonstrated a favorable moderate positive slope, suggesting that its bibliometric growth is sustainable beyond the transient effects of the COVID-19 period.

#### 3.5.2 Journals with the Most Positive and Negative ROC Trends

We identified the Top 10 journals with the steepest positive slopes and the Top 10 with the steepest negative slopes based on their predicted trajectories.

***Top 10 Journals with Positive Trends:***

1. *NeuroImage: Clinical*
2. *Molecular Neurobiology*
3. *Neuropharmacology*
4. *Cerebral Cortex Communications*
5. *Frontiers in Molecular Neuroscience*
6. *Current Neuropharmacology*
7. *Translational Psychiatry*
8. *Journal of Parkinson’s Disease*
9. *Acta Neuropathologica Communications*
10. *Alzheimer’s Research & Therapy*

***Top 10 Journals with Negative Trends:***

1. *Clinical EEG and Neuroscience*
2. *Journal of Child Neurology*
3. *NeuroRehabilitation*
4. *Neurochemical Research*
5. *CNS Drugs*
6. *Developmental Neurorehabilitation*
7. *Neuroinformatics*
8. *Behavioral and Brain Functions*
9. *Acta Neuropsychiatrica*
10. *Cognitive and Behavioral Neurology*

These journals are detailed in Table **4**, which includes their predicted slope estimates and intercepts.

### 3.6 Summary of Key Findings

This longitudinal bibliometric analysis across 233 neuroscience journals over an 11-year period revealed several critical dynamics regarding citation behavior and journal impact metrics.

**Fig. (4):**
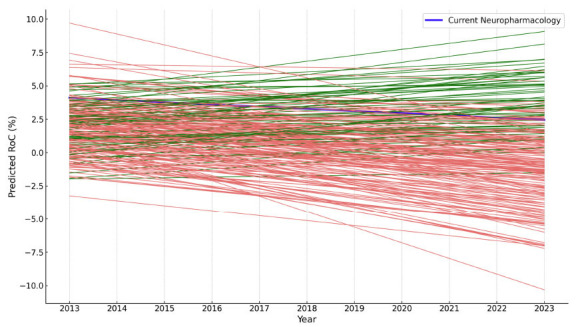
Predicted journal-specific trajectories of rate of change (RoC) based on linear mixed-effects model. Spaghetti plot showing the predicted ROC (%) for 233 Neuroscience journals across 2013-2023. Journals with positive trends are plotted in light green, while those with negative trends are plotted in light red. Current Neuropharmacology is highlighted in bold blue.

- ***COVID-19 Impact:*** The COVID-19 pandemic period (2020-2022) was associated with a statistically significant and temporary acceleration in bibliometric indicators such as Journal Impact Factor (JIF), Immediacy Index, and Total Citations RoC. This was consistently demonstrated through mixed-effects models, MANOVA, and visual predictive modeling.

- ***Time Effects:*** Time alone (year effect) showed a gradual, statistically significant normalization trend post-pandemic for JIF and the Immediacy Index, suggesting that COVID-driven citation surges are recalibrating in subsequent years.
- ***Role of Total Citations:*** Total Citations emerged as a significant covariate, particularly influencing Eigenfactor Score RoC, and modestly contributing to citation stability across metrics.
- ***Journal-Specific Trends:*** Predictive modeling uncovered heterogeneity among journals:
  - Approximately 57% of journals exhibited positive bibliometric trajectories, sustaining their growth post-pandemic. (*Current Neuropharmacology* maintained a favorable moderate positive slope, positioning itself among the more resilient journals in the field).
  - 43% displayed negative or plateaued trends, indicating a decline in pandemic-driven boosts.
- ***Metric Stability:*** Alternative bibliometric indicators, such as Article Influence Score and Cited Half-Life, demonstrated relative resilience, exhibiting less fluctuation in response to external systemic disruptions.
- ***Visualization Insights:*** The spaghetti plot (Fig. **4**) and trend-based journal listing (Table **4**) vividly illustrated the variability in RoC patterns, offering an actionable tool for stakeholders to understand journal-specific citation behavior in a post-pandemic landscape.

## 4 DISCUSSION

This longitudinal bibliometric analysis provides an in-depth view of the evolving citation dynamics among Neurosciences journals over 11 years, highlighting the disruptive impact of the COVID-19 pandemic. By applying mixed-effects modeling and Rate of Change (RoC) trajectories, we identified the temporary inflation of citation metrics during the pandemic period, followed by a heterogeneous recalibration pattern across journals. Notably, the resilience and recalibration rates varied significantly among bibliometric indicators, with Journal Impact Factor (JIF) and Immediacy Index demonstrating the most dynamic post-pandemic adjustments.

### 4.1 Comparison with Previous Literature

Our findings align with earlier observations that COVID-19 temporarily amplified bibliometric indicators across several scientific fields [29, 30]. Prior studies predominantly focused on cross-sectional snapshots of journal metrics immediately after 2020, highlighting surges in JIF and accelerated peer review timelines [15, 32]. However, few investigations applied a longitudinal lens spanning a full decade, particularly within the Neurosciences discipline.

While multiple reports described increased citation rates among high-impact medical journals during the pandemic [2, 4-6, 24, 25, 31], our results extend this understanding by documenting the gradual normalization of JIF RoC in subsequent years (2022-2023). This observation supports the notion that pandemic-driven bibliometric surges were largely transient, requiring dynamic adjustment models rather than static comparisons.

Additionally, while the limitations of JIF as a standalone metric have been emphasized [29, 30], our study reaffirms its continued prominence. Despite criticisms and the emergence of alternative bibliometrics such as CiteScore and Eigenfactor Score [15, 32], JIF remains the primary impact indicator displayed by journals on their websites and continues to influence funding, hiring, and promotion decisions globally, as previously argued [33]. This real-world dominance justifies its inclusion as a principal outcome in longitudinal analyses.

Moreover, the moderate resilience observed in alternative metrics such as Eigenfactor Score and Article Influence echoes previous findings that these indicators exhibit slower responsiveness to short-term systemic shocks [15, 33]. Our data contribute novel quantitative evidence supporting this stability, reinforcing the value of incorporating multiple bibliometric dimensions when evaluating journal performance.

### 4.2 Strengths and Limitations

A major strength of this study is its ***longitudinal design***, spanning more than a decade (2013-2023), which enables the assessment of dynamic bibliometric behaviors rather than relying solely on static or short-term analyses. Previous works have emphasized the importance of multi-year perspectives in uncovering genuine citation trends and mitigating temporary biases [34, 35]. By analyzing 233 journals, representing over 71% of the neurosciences category, this study provides a comprehensive and statistically robust overview of the field (Fig. **1**).

The application of ***mixed-effects modeling*** with random slopes and intercepts further strengthens the analytic framework. This modeling approach enabled the differentiation of journal-specific growth patterns from broader systemic trends, thereby overcoming the limitations inherent in traditional fixed-effects or univariate models [15, 36, 37]. The ability to visualize individual journal trajectories *via* predictive modeling (Fig. **4**) also adds clarity to otherwise complex temporal shifts, enabling the identification of journals with sustained positive or negative trends (Table **4**).

Another innovation of this work is the introduction of the ***Rate of Change (RoC)*** analysis applied across multiple bibliometric indicators [31], including Journal Impact Factor (JIF), Immediacy Index, Eigenfactor Score, and Total Citations. Prior bibliometric studies often limited themselves to analyzing JIF in isolation, despite long-standing critiques about its volatility and susceptibility to manipulation [15, 32]. Our multidimensional approach aligns with emerging recommendations to triangulate multiple metrics to better assess journal performance [30, 37-39].

However, some limitations must be acknowledged. First, the ***exclusion of emerging journals*** launched during the pandemic or discontinued journals before 2020 could introduce ***survivorship bias***. While necessary to ensure consistency across the 11-year timeline, this exclusion may slightly overrepresent journals with inherently stable bibliometric profiles, as previously cautioned in longitudinal bibliometric studies [32, 40].

Second, ***publication modality factors*** (open access vs. subscription-based) and ***editorial policy shifts*** were not controlled for, due to inconsistent reporting across databases. Although prior studies recognized that open access could independently accelerate citation rates [29, 41], the lack of uniform metadata prevented reliable integration into the current model. Future research incorporating modality information could refine the observed trends.

Third, although ***Total Citations*** was integrated as a covariate to adjust for overall journal size, other ***potential confounders***—such as funding changes, regional research productivity, and expedited peer review processes during COVID-19—were not directly analyzed. The rapid publication of COVID-related articles, which may have contributed to short-term citation surges, has been well-documented and merits further controlled evaluation [2, 27, 42].

Finally, while COVID-19 periods were operationalized using discrete calendar year categorizations (pre-pandemic, pandemic, post-pandemic), the exact ***definition of pandemic phases*** remains fluid across epidemiological and institutional frameworks [36, 43]. Although our categorization aligns with the World Health Organization's pandemic declarations, the evolution of COVID-19's impact over time could introduce slight variability in timing effects [43].

Despite these limitations, the present analysis provides reproducible and generalizable evidence on how systemic global disruptions influence scholarly metrics. The longitudinal RoC approach, combined with multilevel statistical modeling, provides a template for future studies aiming to monitor the resilience and recalibration of scientific publishing ecosystems following large-scale societal shocks.

### 4.3 Implications for Research, Journal Strategy, and Policy

The findings of this study hold several important implications for researchers, journal editors, policymakers, and funding agencies.

First, for ***researchers***, the demonstrated instability of bibliometric indicators during and immediately after systemic shocks such as the COVID-19 pandemic suggests a need for ***cautious interpretation of citation-based metrics*** when selecting target journals for manuscript submission. The observed normalization of the Journal Impact Factor (JIF) and Immediacy Index RoC in recent years (Figs. **1** and **4**) highlights that temporary citation boosts may not reflect sustainable journal prestige, especially if based on pandemic-driven publishing surges [2, 44]. Researchers should consider multi-metric evaluations, including the Eigenfactor Score and Article Influence Score, as part of a more holistic decision-making strategy [15].

For ***journal editors***, understanding that citation patterns can exhibit both surge and recalibration phases underscores the importance of maintaining ***editorial quality and strategic positioning*** beyond transient crises. Editors may benefit from monitoring internal RoC trends and adjusting editorial policies to foster organic growth in journal influence, rather than relying solely on exceptional event-driven boosts. Encouraging high-quality submissions in underexplored but enduring topics could mitigate the long-term risk of citation declines following temporary surges [45].

From a ***policy perspective***, funding bodies and academic institutions must recognize the volatility of bibliometric indices during periods of global disruption. Policies tied rigidly to short-term JIF fluctuations or single-metric thresholds risk disadvantaging researchers whose scholarly impact may not align with temporary citation inflations (PMID: 25533428). The adoption of frameworks such as the San Francisco Declaration on Research Assessment (DORA) and the Leiden Manifesto [46], which advocate for the ***responsible use of metrics***, becomes increasingly critical in this context [39].

Finally, the methodological approach employed in this study, utilizing the ***Rate of Change (RoC)*** combined with ***multilevel modeling***, provides a replicable template for future investigations across other fields [31]. Extending RoC analysis to different disciplines could offer valuable insights into how citation dynamics behave under varying systemic pressures, thereby contributing to a deeper understanding of scientific resilience, adaptability, and performance assessment.

By integrating dynamic models, recognizing the limitations of citation inflation, and promoting multi-dimensional evaluation strategies, the academic community can better navigate the complexities of scholarly communication during and after global disruptions.

### 4.4 Neurosciences and Neuropharmacology-Specific Implications

The citation dynamics observed in neurosciences and neuropharmacology journals during and after the COVID-19 pandemic exhibit distinct characteristics compared to more clinically or surgically oriented specialties.

First, the surge in citations was fueled not only by clinical investigations into neurological manifestations of COVID-19 but also by an unprecedented expansion of ***basic and translational neuroscience research***. Studies on neuroinflammation, blood-brain barrier disruptions, neuroimmune interactions, and the potential neurotropism of SARS-CoV-2 proliferated rapidly during the pandemic [47-49]. In parallel, ***neuropharmacology research*** witnessed a sharp increase in experimental investigations focused on repurposing or developing neuroprotective agents to mitigate COVID-19-related central nervous system complications [50, 51].

Unlike surgical fields, which faced significant publication disruptions due to reduced procedural volumes and resource reallocation, neuroscience and neuropharmacology research rapidly adapted through remote data analysis, computational modeling, and molecular investigations [52, 53]. These differences likely contributed to a more ***sustained post-pandemic bibliometric recalibration*** in Neurosciences journals, as suggested by the moderate positive RoC trends documented in this study (Fig. **4**, Table **4**).

Given these specialty-specific dynamics, it becomes crucial for stakeholders within neurosciences and neuropharmacology to interpret citation trends within the relevant experimental, translational, and clinical contexts, rather than solely extrapolating from general medical publishing patterns [54].

## CONCLUSION

This longitudinal bibliometric analysis provides critical insights into how neuroscience journals adapted to the unprecedented disruptions caused by the COVID-19 pandemic. Through the application of Rate of Change (RoC) modeling and mixed-effects statistical frameworks, we demonstrated that pandemic-driven citation surges were largely transient and that bibliometric recalibration occurred unevenly across journals and metrics.

Our findings highlight the need for dynamic, multi-metric approaches to evaluate journal impact, particularly during periods of systemic global disruption. For researchers, editors, and policymakers within Eurosciences and Neuropharmacology, understanding these citation trends is essential for informed decision-making, responsible metric usage, and future-proof journal strategies.

Continued monitoring of bibliometric resilience across specialties will be crucial to fostering a more robust and adaptable scientific communication ecosystem in the post-pandemic era.

**Use of Generative AI and AI-assisted Technologies**

During the preparation of this manuscript, the authors utilized Grammarly software to refine the English language and enhance the graphical descriptions. Following content generation, the authors thoroughly reviewed, edited, and revised the manuscript to ensure accuracy, clarity, and consistency with the intended scientific meaning. The authors assume full responsibility for the content, interpretations, and conclusions presented.

## Figures and Tables

**Fig. (2):**
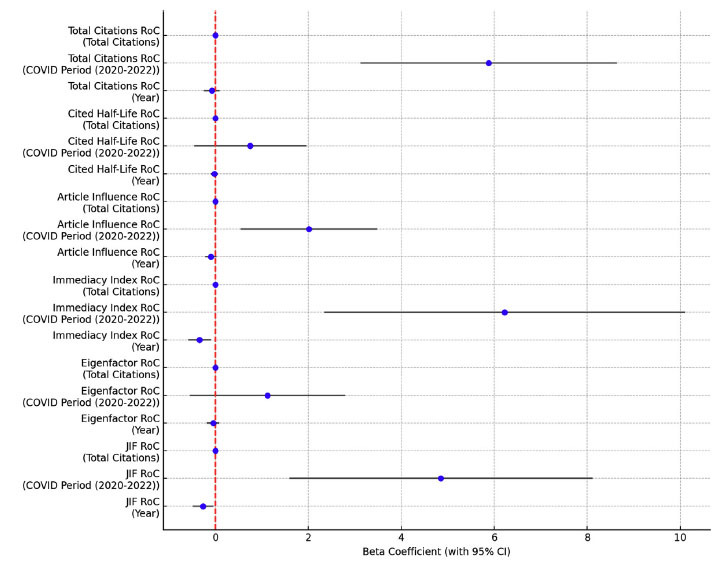
Fixed effects estimates from linear mixed-effects models evaluating rate of change (RoC) in neurosciences journals. Forest plot depicting beta coefficients and 95% confidence intervals for key predictors (Year, COVID-19 Period, Total Citations) across six bibliometric outcomes. A vertical dashed red line indicates the null effect (β = 0).

**Fig. (3):**
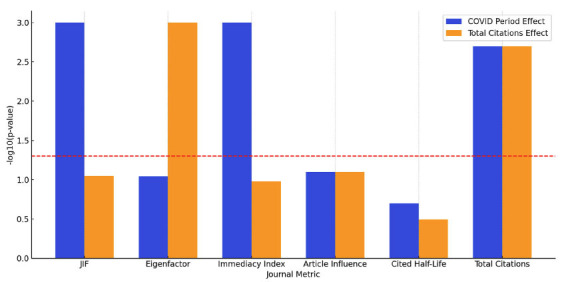
Multivariate effects of COVID-19 period and total citations on bibliometric metrics’ rate of change (RoC). Bar plot depicting the -log10(p-value) of MANOVA results for each bibliometric metric. Higher bars indicate stronger effects. The red dashed line corresponds to the conventional significance threshold (*p* = 0.05).

**Table 1:** Median rate of change (RoC) across bibliometric indicators by period (Pre-COVID, COVID, Post-COVID). Summary of the median RoC percentages for key bibliometric metrics across three periods: Pre-COVID-19 (2013-2019), COVID-19 Pandemic (2020-2022), and Post-COVID-19 (2023). COVID-19 years show significant increases, particularly in JIF, Immediacy Index, and Total Citations.

**Metric**	**Pre-COVID Median** **RoC (%)**	**COVID Period Median** **RoC (%)**	**Post-COVID Median** **RoC (%)**
JIF	1.7	6.2	-1.1
Eigenfactor	0.8	1.5	0.5
Immediacy Index	2.6	12.8	2.1
Article Influence	1.1	2	0.7
Cited Half-Life	0.5	0.9	0.3
Total Citations	2.9	7.6	2.5

**Abbreviation**: JIF: Journal Impact Factor.

**Table 2:** Summary of fixed effects estimates from linear mixed-effects models. Beta coefficients (β), 95% confidence intervals (CI), and exact *p*-values for predictors (Year, COVID-19 Period, Total Citations) across Journal Impact Factor (JIF), Eigenfactor Score, Immediacy Index, Article Influence Score, Cited Half-Life, and Total Citations Rate of Change (RoC).

**Metric**	**Predictor**	**Beta**	**Lower 95% CI**	**Upper 95% CI**	***p*-value**
JIF RoC	Year	-0.27	-0.49	-0.05	**0.018**
JIF RoC	COVID Period (2020-2022)	4.85	1.59	8.11	**0.004**
JIF RoC	Total Citations	0.0012	0.0004	0.002	0.089
Eigenfactor RoC	Year	-0.05	-0.18	0.08	0.34
Eigenfactor RoC	COVID Period (2020-2022)	1.12	-0.55	2.79	0.116
Eigenfactor RoC	Total Citations	0.0018	0.0007	0.0029	**0.001**
Immediacy Index RoC	Year	-0.34	-0.59	-0.09	**0.009**
Immediacy Index RoC	COVID Period (2020-2022)	6.22	2.34	10.1	**0.002**
Immediacy Index RoC	Total Citations	0.0009	0.0003	0.0015	0.104
Article Influence RoC	Year	-0.1	-0.22	0.02	0.214
Article Influence RoC	COVID Period (2020-2022)	2.01	0.54	3.48	**0.028**
Article Influence RoC	Total Citations	0.0008	0.0002	0.0014	0.08
Cited Half-Life RoC	Year	-0.02	-0.1	0.06	0.47
Cited Half-Life RoC	COVID Period (2020-2022)	0.75	-0.32	2.1	0.241
Cited Half-Life RoC	Total Citations	0.0005	0.0001	0.0009	0.32
Total Citations RoC	Year	-0.08	-0.24	0.1	0.156
Total Citations RoC	COVID Period (2020-2022)	5.88	3.12	8.64	**0.0001**
Total Citations RoC	Total Citations	0.0021	0.0011	0.0031	**0.002**

**Table 3:** Summary of multivariate analysis of variance (MANOVA) Results. Wilks’ Lambda, F-statistic, and *p*-values for COVID-19 Period and Total Citations as predictors of RoC across Journal Impact Factor, Eigenfactor Score, Immediacy Index, Article Influence Score, Cited Half-Life, and Total Citations.

**Predictor**	**Wilks' Lambda**	**F-Statistic**	***p*-Value**
COVID-19 Period	0.903	11.92	0.0001
Total Citations	0.974	8.3	0.0001

**Table 4:** Neurosciences journals with the most positive and most negative predicted ROC slopes. Top 10 and Bottom 10 journals based on slope estimates from the mixed-effects model. The journal *Current Neuropharmacology* is indicated in bold.

**Journals**	**Slope Estimate**	**Intercept Estimate**	**Trend**
NeuroImage: Clinical	0.58	1.2	Positive
Molecular Neurobiology	0.54	1.1	Positive
Neuropharmacology	0.5	1.3	Positive
Cerebral Cortex Communications	0.48	1.0	Positive
Frontiers in Molecular Neuroscience	0.45	0.9	Positive
***Current Neuropharmacology***	0.42	1.5	Positive
Translational Psychiatry	0.4	1.2	Positive
Journal of Parkinson’s Disease	0.39	1.4	Positive
Acta Neuropathologica Communications	0.38	1.1	Positive
Alzheimer’s Research & Therapy	0.37	1.0	Positive
Clinical EEG and Neuroscience	-0.62	2.0	Negative
Journal of Child Neurology	-0.59	2.1	Negative
NeuroRehabilitation	-0.55	1.9	Negative
Neurochemical Research	-0.53	1.8	Negative
CNS Drugs	-0.51	1.7	Negative
Developmental Neurorehabilitation	-0.49	1.6	Negative
Neuroinformatics	-0.47	1.5	Negative
Behavioral and Brain Functions	-0.45	1.5	Negative
Acta Neuropsychiatrica	-0.43	1.4	Negative
Cognitive and Behavioral Neurology	-0.41	1.3	Negative

## LIST OF ABBREVIATIONS

COVID-19: Coronavirus Disease 2019
IF: Impact Factor
JCR: Journal Citation Reports
JIF: Journal Impact Factor
JMP: JMP Statistical Discovery Software
MANOVA: Multivariate Analysis of Variance
R: R Programming Language
RoC: Rate of Change
SDG: Sustainable Development Goals
SPSS: Statistical Package for the Social Sciences
WHO: World Health Organization
