# Supplementary material for: Resilience and Recalibration of Bibliometric Indicators in Neurosciences and Neuropharmacology Journals After COVID-19: A Longitudinal Rate of Change Analysis Using Mixed-Effects Models
Source: Curr Neuropharmacol. 2025 Jul 21;24(5):612–24. doi: 10.2174/011570159X384613250702110845 (PMC13269942; doi:10.2174/011570159X384613250702110845)
Supplement: Supplementary file 1 — Supplementary material is available on the publisher’s website along with the published article. [file CN-24-5-612_SD1.pdf]

Supplementary Material

Resilience and Recalibration of Bibliometric Indicators in Neurosciences and Neuropharmacology Journals After COVID-19: A Longitudinal Rate of Change Analysis Using Mixed-Effects Models

Camilo Rios-Castañeda[1], Heriberto Aguirre-Meneses[2], Marco-Antonio Nuñez-Gaona[2] and Ernesto Roldan-Valadez[1,3,*]

[1]*Division of Neurosciences, Instituto Nacional de Rehabilitacion 'Luis Guillermo Ibarra Ibarra', 14389, Mexico City, Mexico;* [2]*Department of Medical Systems, Instituto Nacional de Rehabilitacion 'Luis Guillermo Ibarra Ibarra', 14389, Mexico City, Mexico;* [3]*I.M. Sechenov First Moscow State Medical University (Sechenov University), Department of Radiology, 119992, Moscow, Russia*

1875-6190/26 $65.00+.00 **© 2026 Bentham Science Publishers**

IF Journal Active 11 Years

Journal Name	Active Years	2022 Impact Factor	2021 Impact Factor	2020 Impact Factor	2019 Impact Factor	2018 Impact Factor	2017 Impact Factor	2016 Impact Factor	2015 Impact Factor	2014 Impact Factor	2013 Impact Factor	2012 Impact Factor
ACS CHEMICAL NEUROSCIENCE	11	5	5.78	4.418	4.486	3.861	4.211	3.883	4.348	4.362	4.21	3.871
ACTA NEUROBIOLOGIAE EXPERIMENTALIS	11	1.4	1.269	1.579	1.541	1.529	1.5	1.207	1.708	1.286	2.244	1.977
ACTA NEUROLOGICA BELGICA	11	2.7	2.471	2.396	1.989	1.612	2.072	1.722	1.495	0.894	0.598	0.466
ACTA NEUROPATHOLOGICA	11	12.7	15.887	17.088	14.256	18.174	15.876	12.213	11.36	10.762	9.777	9.734
ACTA NEUROPSYCHIATRICA	11	3.8	4.513	3.403	3	1.978	2.333	1.939	0.76	0.802	0.639	0.606
ACTAS ESPANOLAS DE PSIQUIATRIA	11	1.5	1.667	1.196	1.681	1.479	0.878	1.339	0.813	1.2	0.759	0.452
ACUPUNCTURE & ELECTRO-THERAPEUTICS RESEARCH	11	0.3	0.684	0.143	0.2	0.417	0.435	0.87	0.778	0.824	0.4	0.737
ALZHEIMERS RESEARCH & THERAPY	11	9	8.831	6.982	6.116	6.142	5.015	6.154	5.197	3.979	3.5	4.39
ANNALS OF NEUROLOGY	11	11.2	11.274	10.422	9.037	9.496	10.25	9.89	9.638	9.977	11.91	11.193
ANNUAL REVIEW OF NEUROSCIENCE	11	13.9	15.553	12.449	12.528	12.043	14.675	15.63	14.265	19.32	22.66	20.614
ARCHIVES ITALIENNES DE BIOLOGIE	11	1	1.619	1	1.179	0.974	0.595	0.58	0.61	1.488	1.422	1.433
ARQUIVOS DE NEURO-PSIQUIATRIA	11	1.4	2.035	1.42	1.008	1.048	1.015	0.902	0.937	0.843	1.006	0.827
ASN NEURO	11	4.7	5.2	4.146	4.167	2.707	3.617	3.03	2.828	4.017	4.436	3.638
AUDIOLOGY AND NEURO-OTOLOGY	11	1.6	2.213	1.854	1.549	2.053	2.078	1.791	1.776	1.705	1.852	2.318
AUTONOMIC NEUROSCIENCE-BASIC & CLINICAL	11	2.7	2.355	3.145	2.2	2.247	2.605	2.225	1.621	1.562	1.372	1.846
BEHAVIORAL AND BRAIN FUNCTIONS	11	5.1	3.95	3.759	2.125	2.457	2.449	2.207	1.72	1.972	2	2.789
BEHAVIORAL AND BRAIN SCIENCES	11	29.3	21.357	12.579	17.333	17.194	15.071	14.2	20.415	20.771	14.962	18.571
BEHAVIORAL NEUROSCIENCE	11	1.9	2.154	1.912	1.714	2.102	2.507	2.453	2.69	2.728	3.25	2.634
BEHAVIOURAL BRAIN RESEARCH	11	2.7	3.352	3.332	2.977	2.77	3.173	3.002	3.002	3.028	3.391	3.327
BEHAVIOURAL PHARMACOLOGY	11	1.6	2.277	2.293	1.741	1.788	1.854	2.218	2	2.148	2.194	2.301
BIOLOGICAL CYBERNETICS	11	1.9	3.072	2.086	1.111	1.305	1.44	1.716	1.611	1.713	1.933	2.067
BIOLOGICAL PSYCHIATRY	11	10.6	12.81	13.382	12.095	11.501	11.984	11.412	11.212	10.255	9.472	9.247
BIPOLAR DISORDERS	11	5.9	5.345	6.744	5.41	4.936	4.49	4.531	4.882	4.965	4.888	4.621
BMC NEUROSCIENCE	11	2.4	3.264	3.288	2.811	2.62	2.173	2.312	2.304	2.665	2.845	3
BRAIN	11	14.5	15.255	13.501	11.337	11.814	10.848	10.292	10.103	9.196	10.226	9.915
BRAIN AND COGNITION	11	2.5	2.682	2.31	2.508	2.619	2.574	2.432	2.399	2.477	2.683	2.823

Journal Name	Active Years	2022 Impact Factor	2021 Impact Factor	2020 Impact Factor	2019 Impact Factor	2018 Impact Factor	2017 Impact Factor	2016 Impact Factor	2015 Impact Factor	2014 Impact Factor	2013 Impact Factor	2012 Impact Factor
BRAIN AND LANGUAGE	11	2.5	2.781	2.381	2.339	2.7	2.851	2.439	3.038	3.215	3.309	3.386
BRAIN BEHAVIOR AND EVOLUTION	11	1.7	1.919	1.808	1.875	1.542	1.65	1.915	2.103	2.013	4.288	2.885
BRAIN BEHAVIOR AND IMMUNITY	11	15.1	19.227	7.217	6.633	6.17	6.306	5.964	5.874	5.889	6.128	5.612
BRAIN IMPAIRMENT	11	0.8	1.14	1.727	1.356	0.958	0.977	0.6	0.565	0.809	0.878	0.65
BRAIN INJURY	11	1.9	2.167	2.311	1.69	1.665	2.061	1.971	1.822	1.808	1.861	1.513
BRAIN PATHOLOGY	11	6.4	7.611	6.508	5.568	6.352	6.187	6.624	5.256	4.643	4.354	4.739
BRAIN RESEARCH	11	2.9	3.61	3.252	2.733	2.929	3.125	2.746	2.561	2.843	2.828	2.879
BRAIN RESEARCH BULLETIN	11	3.8	3.715	4.079	3.37	3.103	3.44	3.033	2.572	2.718	2.974	2.935
BRAIN STIMULATION	11	7.7	9.184	8.955	6.565	6.919	6.12	6.078	4.793	4.399	5.432	4.538
BRAIN STRUCTURE & FUNCTION	11	3.1	3.748	3.27	3.298	3.622	4.231	4.698	5.811	5.618	4.567	7.837
BRAIN TOPOGRAPHY	11	2.7	4.275	3.02	2.759	3.104	2.703	3.394	3.727	3.468	2.519	3.671
CELLULAR AND MOLECULAR NEUROBIOLOGY	11	4	4.231	5.046	3.606	3.811	3.895	2.939	2.328	2.506	2.201	2.293
CEPHALALGIA	11	4.9	6.075	6.295	4.868	4.438	3.886	3.609	6.052	4.891	4.121	3.485
CEREBELLUM	11	3.5	3.648	3.847	3.129	3.413	3.199	3.234	2.429	2.717	2.864	2.595
CEREBRAL CORTEX	11	3.7	4.861	5.357	5.043	5.437	6.308	6.559	8.285	8.665	8.305	6.828
CESKA A SLOVENSKA NEUROLOGIE A NEUROCHIRURGIE	11	0.5	0.411	0.35	0.377	0.355	0.508	0.368	0.209	0.165	0.159	0.372
CHEMICAL SENSES	11	4.2	4.985	3.16	2.261	2.336	3.235	2.52	2.5	3.157	3.278	3.222
CHEMOSENSORY PERCEPTION	11	1	1.323	1.833	2	0.824	1.641	1.474	1.053	1.302	1.365	1.317
CLINICAL AUTONOMIC RESEARCH	11	5.8	5.625	4.435	2.968	2.485	1.635	1.276	1.257	1.487	1.864	1.478
CLINICAL EEG AND NEUROSCIENCE	11	2	2.046	1.843	1.765	1.822	1.807	2.163	1.862	2.219	3.157	1.818
CLINICAL NEUROPHYSIOLOGY	11	4.7	4.861	3.708	3.214	3.675	3.614	3.866	3.426	3.097	2.979	3.144
CNS NEUROSCIENCE & THERAPEUTICS	11	5.5	7.035	5.243	4.074	3.394	3.495	3.919	4.019	3.931	3.784	4.458
COGNITIVE AFFECTIVE & BEHAVIORAL NEUROSCIENCE	11	2.9	3.526	3.282	2.206	2.661	2.565	3.263	2.886	3.287	3.209	3.866
COGNITIVE COMPUTATION	11	5.4	4.89	5.418	4.307	4.287	3.479	3.441	1.933	1.44	1.1	0.867
COGNITIVE NEURODYNAMICS	11	3.7	3.473	5.082	3.925	3.021	2	1.828	2.159	1.671	1.77	1.742
COGNITIVE NEUROSCIENCE	11	2	2.55	3.065	3	3.361	3.417	1.87	2.373	2.653	2.375	2.192
COGNITIVE SYSTEMS RESEARCH	11	3.9	4.541	3.523	1.902	1.384	1.425	1.182	1.204	0.831	0.754	0.75

Journal Name	Active Years	2022 Impact Factor	2021 Impact Factor	2020 Impact Factor	2019 Impact Factor	2018 Impact Factor	2017 Impact Factor	2016 Impact Factor	2015 Impact Factor	2014 Impact Factor	2013 Impact Factor	2012 Impact Factor
CORTEX	11	3.6	4.644	4.027	4.009	4.275	4.907	4.279	4.314	5.128	6.042	6.161
CURRENT ALZHEIMER RESEARCH	11	2.1	3.04	3.498	3.047	3.271	3.289	2.952	3.145	3.889	3.796	3.676
CURRENT NEUROLOGY AND NEUROSCIENCE REPORTS	11	5.6	6.03	5.081	4.376	3.4	3.478	3.345	2.961	3.059	3.669	3.783
CURRENT NEUROPHARMACOLOGY	11	5.3	7.708	7.39	4.668	4.568	4.068	3.365	3.753	3.049	2.347	2.031
CURRENT NEUROVASCULAR RESEARCH	11	2.1	2.294	1.99	1.649	1.811	1.716	2.298	2.123	2.253	2.735	2.844
CURRENT OPINION IN NEUROBIOLOGY	11	5.7	7.07	6.627	6.263	6.014	6.541	6.133	6.373	6.628	6.765	7.335
CURRENT OPINION IN NEUROLOGY	11	4.8	6.283	5.71	4.207	4.647	4.01	4.699	4.469	5.307	5.729	5.416
DEVELOPMENTAL COGNITIVE NEUROSCIENCE	11	4.7	5.811	6.464	4.966	4.92	4.815	4.321	3.963	3.833	3.705	3.16
DEVELOPMENTAL NEUROBIOLOGY	11	3	3.102	3.964	3.935	2.6	2.598	2.972	2.529	3.37	4.189	4.423
DEVELOPMENTAL NEUROSCIENCE	11	2.9	3.421	2.984	3.041	2.125	2.133	3.033	2.898	2.697	2.453	3.413
ENCEPHALE-REVUE DE PSYCHIATRIE CLINIQUE BIOLOGIQUE ET THERAPEUTIQUE	11	2.7	2.787	1.291	0.873	0.865	0.599	0.742	0.675	0.698	0.598	0.493
EUROPEAN JOURNAL OF NEUROLOGY	11	5.1	6.288	6.089	4.516	4.387	4.621	3.988	3.956	4.055	3.852	4.162
EUROPEAN JOURNAL OF NEUROSCIENCE	11	3.4	3.698	3.386	3.115	2.784	2.832	2.941	2.975	3.181	3.669	3.753
EUROPEAN JOURNAL OF PAIN	11	3.6	3.651	3.934	3.492	3.188	2.991	3.019	2.9	2.928	3.218	3.067
EUROPEAN NEUROLOGY	11	2.4	2.292	1.71	1.182	1.235	1.562	1.697	1.403	1.356	1.362	1.5
EUROPEAN NEUROPSYCHOPHARMACOLOGY	11	5.6	5.415	4.6	3.853	4.468	4.129	4.239	4.409	4.369	5.395	4.595
EXPERIMENTAL BRAIN RESEARCH	11	2	2.064	1.972	1.591	1.878	1.806	1.917	2.057	2.036	2.168	2.221
EXPERIMENTAL NEUROLOGY	11	5.3	5.62	5.33	4.691	4.562	4.483	4.706	4.657	4.696	4.617	4.645
FOLIA NEUROPATHOLOGICA	11	2	2.494	2.038	1.278	1.16	1.345	1.093	1.233	1.568	1.667	1.547
FRONTIERS IN AGING NEUROSCIENCE	11	4.8	5.702	5.75	4.364	3.633	3.582	4.504	4.348	4	2.843	5.224
FRONTIERS IN BEHAVIORAL NEUROSCIENCE	11	3	3.617	3.558	2.512	2.622	3.138	3.104	3.392	3.27	4.16	4.758
FRONTIERS IN CELLULAR NEUROSCIENCE	11	5.3	6.147	5.505	3.921	3.9	4.3	4.555	4.609	4.289	4.175	4.469
FRONTIERS IN COMPUTATIONAL NEUROSCIENCE	11	3.2	3.387	2.38	2.535	2.323	2.073	1.821	2.653	2.201	2.233	2.481
FRONTIERS IN HUMAN NEUROSCIENCE	11	2.9	3.473	3.169	2.673	2.87	2.871	3.209	3.634	3.626	2.895	2.906
FRONTIERS IN NEURAL CIRCUITS	11	3.5	3.342	3.492	3.156	3.101	3.131	3.005	3.879	3.568	2.95	3.333
FRONTIERS IN NEUROANATOMY	11	2.9	3.543	3.856	3.292	2.923	3.152	3.267	3.26	3.544	4.176	4.058
FRONTIERS IN NEUROENDOCRINOLOGY	11	7.4	8.333	8.606	9.044	7.852	6.875	9.425	8.852	7.037	7.581	7.985

Journal Name	Active Years	2022 Impact Factor	2021 Impact Factor	2020 Impact Factor	2019 Impact Factor	2018 Impact Factor	2017 Impact Factor	2016 Impact Factor	2015 Impact Factor	2014 Impact Factor	2013 Impact Factor	2012 Impact Factor
GAIT & POSTURE	11	2.4	2.746	2.84	2.349	2.414	2.273	2.347	2.286	2.752	2.299	1.969
GENES BRAIN AND BEHAVIOR	11	2.5	3.708	3.449	3.397	3.157	3.496	3.743	3.275	3.661	3.505	3.597
GLIA	11	6.2	8.073	7.452	5.984	5.829	5.846	6.2	5.997	6.031	5.466	5.066
HEARING RESEARCH	11	2.8	3.672	3.208	3.693	2.952	2.824	2.906	3.565	2.968	2.848	2.537
HIPPOCAMPUS	11	3.5	3.753	3.899	3.404	3.267	3.966	3.945	4.074	4.162	4.302	5.492
HUMAN BRAIN MAPPING	11	4.8	5.399	5.038	4.421	4.554	4.927	4.53	4.962	5.969	6.924	6.878
HUMAN MOVEMENT SCIENCE	11	2.1	2.397	2.161	2.096	1.928	1.84	1.841	1.606	1.598	2.027	2.064
IDEGGYOGYASZATI SZEMLE-CLINICAL NEUROSCIENCE	11	0.8	0.708	0.427	0.337	0.113	0.252	0.322	0.376	0.386	0.343	0.348
INTERNATIONAL JOURNAL OF DEVELOPMENTAL NEUROSCIENCE	11	1.8	2.54	2.457	1.911	2.367	2.495	2.046	2.38	2.58	2.918	2.692
INTERNATIONAL JOURNAL OF NEUROPSYCHOPHARMACOLOGY	11	4.8	5.678	5.176	4.333	4.207	3.981	4.712	4.333	4.009	5.264	5.641
INTERNATIONAL JOURNAL OF NEUROSCIENCE	11	2.2	2.59	2.292	2.107	1.852	1.848	1.75	1.717	1.521	1.528	1.216
INTERNATIONAL JOURNAL OF PSYCHOPHYSIOLOGY	11	3	2.903	2.997	2.631	2.407	2.868	2.582	2.596	2.882	2.648	2.036
JARO-JOURNAL OF THE ASSOCIATION FOR RESEARCH IN OTOLARYNGOLOGY	11	2.4	3.263	3.057	2.76	2.716	2.716	2.455	3.03	2.598	2.547	2.952
JOURNAL OF ALZHEIMERS DISEASE	11	4	4.16	4.472	3.909	3.517	3.476	3.731	3.92	4.151	3.612	4.174
JOURNAL OF CEREBRAL BLOOD FLOW AND METABOLISM	11	6.3	6.597	6.2	5.681	6.04	6.045	5.081	4.929	5.407	5.339	5.398
JOURNAL OF CHEMICAL NEUROANATOMY	11	2.8	3.097	3.052	2.353	2.357	2.162	1.925	1.8	1.5	2.52	2.475
JOURNAL OF CLINICAL NEUROPHYSIOLOGY	11	2.4	2.59	2.177	1.434	1.673	1.982	1.224	1.337	1.429	1.595	1.447
JOURNAL OF CLINICAL NEUROSCIENCE	11	2	2.116	1.961	1.76	1.593	1.64	1.557	1.387	1.378	1.318	1.253
JOURNAL OF COGNITIVE NEUROSCIENCE	11	3.2	3.42	3.225	3.105	3.029	3.468	3.108	3.559	4.085	4.687	4.493
JOURNAL OF COMPARATIVE NEUROLOGY	11	2.5	3.028	3.215	2.801	3.239	3.4	3.266	3.331	3.225	3.508	3.661
JOURNAL OF COMPARATIVE PHYSIOLOGY A-NEUROETHOLOGY SENSORY NEURAL AND BEHAVIORAL PHYSIOLOGY	11	2.1	2.389	1.836	1.516	1.882	1.97	2.429	1.988	2.036	1.634	1.856
JOURNAL OF COMPUTATIONAL NEUROSCIENCE	11	1.2	1.453	1.621	1.811	1.568	1.606	1.483	1.871	1.739	2.087	2.439
JOURNAL OF ELECTROMYOGRAPHY AND KINESIOLOGY	11	2.5	2.641	2.368	1.74	1.753	1.568	1.51	1.53	1.647	1.725	1.644

Journal Name	Active Years	2022 Impact Factor	2021 Impact Factor	2020 Impact Factor	2019 Impact Factor	2018 Impact Factor	2017 Impact Factor	2016 Impact Factor	2015 Impact Factor	2014 Impact Factor	2013 Impact Factor	2012 Impact Factor
JOURNAL OF HEADACHE AND PAIN	11	7.4	8.588	7.277	4.797	3.918	3.403	3.58	3.497	2.801	3.281	2.779
JOURNAL OF INTEGRATIVE NEUROSCIENCE	11	1.8	1.664	2.117	1.193	1.139	0.662	0.647	0.791	0.935	1.121	1.146
JOURNAL OF MOLECULAR NEUROSCIENCE	11	3.1	2.866	3.444	2.678	2.577	2.454	2.229	2.352	2.343	2.757	2.891
JOURNAL OF MOTOR BEHAVIOR	11	1.4	1.358	1.328	1.279	1.313	1.513	1.327	1.573	1.418	1.406	1.042
JOURNAL OF MUSCULOSKELETAL & NEURONAL INTERACTIONS	11	1.9	1.864	2.041	1.66	1.562	1.651	1.489	1.638	1.744	2.4	2.453
JOURNAL OF NEURAL ENGINEERING	11	4	5.043	5.379	4.141	4.551	3.92	3.465	3.493	3.295	3.415	3.282
JOURNAL OF NEURAL TRANSMISSION	11	3.3	3.85	3.575	3.505	2.903	2.779	2.392	2.587	2.402	2.871	3.052
JOURNAL OF NEUROCHEMISTRY	11	4.7	5.546	5.372	4.066	4.87	4.609	4.083	3.842	4.281	4.244	3.973
JOURNAL OF NEURODEVELOPMENTAL DISORDERS	11	4.9	4.074	4.025	3.487	3.59	3.5	3.582	2.787	3.268	3.705	3.448
JOURNAL OF NEUROENDOCRINOLOGY	11	3.2	3.87	3.627	2.886	3.04	3.392	3.47	3.172	3.138	3.507	3.331
JOURNAL OF NEUROENGINEERING AND REHABILITATION	11	5.1	5.208	4.262	3.519	3.582	3.865	3.516	2.419	2.74	2.622	2.567
JOURNAL OF NEUROGENETICS	11	1.9	1.696	1.25	1.438	1.698	1.536	2.291	1.854	1.268	1.383	2.159
JOURNAL OF NEUROIMMUNE PHARMACOLOGY	11	6.2	7.285	4.147	4.113	3.87	3.664	3.339	3.896	4.11	3.172	3.802
JOURNAL OF NEUROIMMUNOLOGY	11	3.3	3.221	3.478	3.125	2.832	2.655	2.72	2.536	2.467	2.786	3.033
JOURNAL OF NEUROINFLAMMATION	11	9.3	9.589	8.322	5.793	5.7	5.193	5.102	4.667	5.408	4.902	4.351
JOURNAL OF NEUROLINGUISTICS	11	2	2.373	1.71	1.853	1.247	1.547	1.403	1.296	1.489	1.596	1.115
JOURNAL OF NEUROPATHOLOGY AND EXPERIMENTAL NEUROLOGY	11	3.2	3.148	3.685	2.923	3.46	3.49	3.503	3.432	3.797	4.372	4.345
JOURNAL OF NEUROPHYSIOLOGY	11	2.5	2.974	2.714	2.234	2.614	2.502	2.396	2.653	2.887	3.041	3.301
JOURNAL OF NEUROPSYCHIATRY AND CLINICAL NEUROSCIENCES	11	2.9	2.891	2.198	2.192	1.971	1.854	1.846	2.433	2.817	2.765	2.397
JOURNAL OF NEUROSCIENCE	11	5.3	6.709	6.167	5.674	6.074	5.971	5.988	5.924	6.344	6.747	6.908
JOURNAL OF NEUROSCIENCE METHODS	11	3	2.987	2.39	2.214	2.785	2.668	2.554	2.053	2.025	1.959	2.114
JOURNAL OF NEUROSCIENCE RESEARCH	11	4.2	4.433	4.164	4.699	4.139	2.662	2.481	2.689	2.594	2.729	2.974
JOURNAL OF NEUROTRAUMA	11	4.2	4.869	5.269	4.056	3.754	5.002	5.19	4.377	3.714	3.968	4.295
JOURNAL OF NEUROVIROLOGY	11	3.2	3.739	2.643	2.354	2.302	3.228	3.206	2.569	2.595	3.323	2.85
JOURNAL OF PAIN	11	4	5.383	5.828	4.621	5.424	4.859	4.519	4.463	4.01	4.216	3.24
JOURNAL OF PARKINSONS DISEASE	11	5.2	5.52	5.568	5.178	3.698	3.172	2.538	3.015	1.91	1.097	1.083

Journal Name	Active Years	2022 Impact Factor	2021 Impact Factor	2020 Impact Factor	2019 Impact Factor	2018 Impact Factor	2017 Impact Factor	2016 Impact Factor	2015 Impact Factor	2014 Impact Factor	2013 Impact Factor	2012 Impact Factor
JOURNAL OF PHYSIOLOGY-LONDON	11	5.5	6.228	5.182	4.547	4.984	4.54	4.739	4.731	5.037	4.544	4.38
JOURNAL OF PINEAL RESEARCH	11	10.3	12.081	13.007	14.528	15.221	11.613	10.391	9.314	9.6	7.812	7.304
JOURNAL OF PSYCHIATRY & NEUROSCIENCE	11	4.3	5.699	6.186	4.382	4.899	5.365	5.165	5.57	5.861	7.492	6.242
JOURNAL OF PSYCHOPHARMACOLOGY	11	4.1	4.562	4.153	3.121	4.221	4.738	4.179	3.637	3.898	3.396	3.374
JOURNAL OF PSYCHOPHYSIOLOGY	11	1.3	1.229	1.333	1.406	1	0.917	0.683	1.167	1.59	1.425	1
JOURNAL OF SLEEP RESEARCH	11	4.4	5.296	3.981	3.623	3.432	3.433	3.259	3.093	3.347	2.949	3.043
JOURNAL OF STROKE & CEREBROVASCULAR DISEASES	11	2.5	2.677	2.136	1.787	1.646	1.598	1.517	1.599	1.669	1.993	1.984
JOURNAL OF THE HISTORY OF THE NEUROSCIENCES	11	0.5	0.644	0.529	0.347	0.244	0.288	0.633	0.529	0.562	0.537	0.282
JOURNAL OF THE INTERNATIONAL NEUROPSYCHOLOGICAL SOCIETY	11	2.6	3.114	2.892	2.576	3.098	2.777	2.181	2.633	2.963	3.009	2.697
JOURNAL OF THE NEUROLOGICAL SCIENCES	11	4.4	4.553	3.181	3.115	2.651	2.448	2.295	2.126	2.474	2.262	2.243
JOURNAL OF THE PERIPHERAL NERVOUS SYSTEM	11	3.8	5.188	3.494	2.466	2.441	2.55	2.361	2.258	2.758	2.504	2.57
JOURNAL OF VESTIBULAR RESEARCH-EQUILIBRIUM & ORIENTATION	11	2.3	2.354	2.435	2.816	2.774	2.865	0.9	1.047	1.19	1.456	1
LEARNING & MEMORY	11	2	2.699	2.46	2.359	2.373	2.671	2.894	2.906	3.657	4.375	4.057
METABOLIC BRAIN DISEASE	11	3.6	3.655	3.584	2.726	2.411	2.441	2.297	2.603	2.638	2.398	2.333
MOLECULAR AND CELLULAR NEUROSCIENCE	11	3.5	4.626	4.314	3.182	2.855	3.312	3.084	3.597	3.84	3.734	3.837
MOLECULAR BRAIN	11	3.6	4.399	4.041	4.686	4.051	3.449	3.41	3.745	4.902	4.345	4.202
MOLECULAR NEUROBIOLOGY	11	5.1	5.686	5.59	4.5	4.586	5.076	6.19	5.397	5.137	5.286	5.471
MOLECULAR NEURODEGENERATION	11	15.1	18.897	14.195	9.599	8.274	6.426	6.78	6.51	6.563	5.286	4.007
MOLECULAR PAIN	11	3.3	3.37	3.395	2.696	2.746	3.205	3.533	3.07	3.654	3.531	3.774
MOLECULAR PSYCHIATRY	11	11	13.437	15.992	12.384	11.973	11.64	13.204	13.314	14.496	15.147	14.897
MOTOR CONTROL	11	1.1	1.535	1.422	1.017	1.302	0.957	0.75	1.037	1.233	1.453	1.39
MUSCLE & NERVE	11	3.4	3.852	3.217	2.505	2.393	2.496	2.605	2.713	2.283	2.311	2.314
NATURE NEUROSCIENCE	11	25	28.771	24.884	20.071	21.126	19.912	17.839	16.724	16.095	14.976	15.251
NATURE REVIEWS NEUROSCIENCE	11	34.7	38.755	34.87	33.654	33.162	32.635	28.88	29.298	31.427	31.376	31.673
NETWORK-COMPUTATION IN NEURAL SYSTEMS	11	7.8	1.5	1.273	0.5	1	0.706	0.562	0.647	0.87	0.5	0.333
NEURAL COMPUTATION	11	2.9	3.278	2.026	2.505	2.261	1.651	1.938	1.626	2.207	1.694	1.76

Journal Name	Active Years	2022 Impact Factor	2021 Impact Factor	2020 Impact Factor	2019 Impact Factor	2018 Impact Factor	2017 Impact Factor	2016 Impact Factor	2015 Impact Factor	2014 Impact Factor	2013 Impact Factor	2012 Impact Factor
NEURAL DEVELOPMENT	11	3.6	3.8	3.842	2.63	2.317	2.13	2.077	3.038	3.453	3.372	3.549
NEURAL NETWORKS	11	7.8	9.657	8.05	5.535	5.785	7.197	5.287	3.216	2.708	2.076	1.927
NEURAL PLASTICITY	11	3.1	3.144	3.599	3.093	3.591	3.161	3.054	3.568	3.582	3.608	2.864
NEURAL REGENERATION RESEARCH	11	6.1	6.058	5.135	3.171	2.472	2.234	1.769	0.968	0.22	0.234	0.144
NEUROBIOLOGY OF AGING	11	4.2	5.133	4.673	4.347	4.398	4.454	5.117	5.153	5.013	4.853	6.166
NEUROBIOLOGY OF DISEASE	11	6.1	7.046	5.996	5.332	5.16	5.227	5.02	4.856	5.078	5.202	5.624
NEUROBIOLOGY OF LEARNING AND MEMORY	11	2.7	3.109	2.877	2.768	3.01	3.244	3.543	3.439	3.652	4.035	3.327
NEUROCHEMICAL JOURNAL	11	0.5	0.448	0.48	0.414	0.298	0.404	0.34	0.29	0.303	0.193	0.235
NEUROCHEMICAL RESEARCH	11	4.4	4.414	3.996	3.038	2.782	2.772	2.581	2.472	2.593	2.551	2.125
NEUROCHEMISTRY INTERNATIONAL	11	4.2	4.297	3.921	3.881	3.994	3.603	3.262	3.385	3.092	2.65	2.659
NEUROCIRUGIA	11	0.8	0.817	0.553	0.597	0.519	0.31	0.548	0.41	0.293	0.322	0.343
NEURODEGENERATIVE DISEASES	11	3	3.417	2.977	2.418	2.798	2.785	2.842	2.937	3.511	3.454	3.41
NEUROENDOCRINOLOGY	11	4.1	5.135	4.914	4.271	6.804	5.024	3.608	2.583	4.373	4.934	3.537
NEUROENDOCRINOLOGY LETTERS	11	0.7	0.638	0.765	0.75	0.698	0.754	0.918	0.946	0.799	0.935	0.932
NEUROGASTROENTEROLOGY AND MOTILITY	11	3.5	3.96	3.598	2.946	3.803	3.842	3.617	3.31	3.587	3.424	2.935
NEUROIMAGE	11	5.7	7.4	6.556	5.902	5.812	5.426	5.835	5.463	6.357	6.132	6.252
NEUROIMAGING CLINICS OF NORTH AMERICA	11	2.3	2.624	2.264	2.632	2.046	1.275	1.325	1.557	1.527	1.289	1.204
NEUROIMMUNOMODULATION	11	2.4	2.795	2.492	1.685	1.351	2.238	2.674	2.361	1.882	1.779	1.835
NEUROINFORMATICS	11	3	2.864	4.085	3.3	5.127	3.852	3.2	2.864	2.825	3.102	3.136
NEUROLOGIC CLINICS	11	2.4	3.787	3.806	2.91	2.802	3.072	2.648	1.973	1.395	1.607	1.342
NEUROLOGICAL RESEARCH	11	1.9	2.529	2.451	2.401	1.983	1.449	1.376	1.418	1.439	1.449	1.182
NEUROLOGICAL SCIENCES	11	3.3	3.83	3.307	2.415	2.484	2.285	1.749	1.783	1.447	1.495	1.412
NEUROLOGY INDIA	11	2.7	1.663	2.117	2.128	2.708	2.166	1.758	1.41	1.232	1.084	1.044
NEUROMOLECULAR MEDICINE	11	3.5	4.103	3.843	2.629	2.576	2.952	3.287	3.692	3.678	3.885	4.492
NEUROMUSCULAR DISORDERS	11	2.8	3.538	4.296	3.115	2.612	2.487	2.969	3.107	2.638	3.134	3.464
NEURON	11	16.2	18.688	17.173	14.415	14.403	14.319	14.024	13.974	15.054	15.982	15.766
NEUROPATHOLOGY	11	2.3	2.076	1.906	1.758	2.161	1.887	1.784	1.556	1.651	1.796	1.909
NEUROPATHOLOGY AND APPLIED NEUROBIOLOGY	11	5	6.25	8.09	7.5	6.878	6.059	5.347	4.483	3.927	4.97	4.837

**Journal Name**	**Active Years**	**2022 Impact Factor**	**2021 Impact Factor**	**2020 Impact Factor**	**2019 Impact Factor**	**2018 Impact Factor**	**2017 Impact Factor**	**2016 Impact Factor**	**2015 Impact Factor**	**2014 Impact Factor**	**2013 Impact Factor**	**2012 Impact Factor**
NEUROPEPTIDES	11	2.9	3.152	3.286	2.411	2.407	2.915	2.486	2.726	2.644	2.546	2.067
NEUROPHARMACOLOGY	11	4.7	5.273	5.251	4.431	4.367	4.249	5.012	4.936	5.106	4.819	4.114
NEUROPHYSIOLOGIE CLINIQUE-CLINICAL NEUROPHYSIOLOGY	11	3	3.1	3.734	2.553	2.167	2.045	1.593	1.479	1.238	1.463	2.553
NEUROPHYSIOLOGY	11	0.5	0.813	0.587	0.322	0.267	0.368	0.207	0.2	0.195	0.174	0.384
NEUROPSYCHOBIOLOGY	11	3.2	12.329	2.328	1.694	1.675	1.421	1.491	1.763	2.261	2.303	2.371
NEUROPSYCHOLOGIA	11	2.6	3.054	3.139	2.652	2.872	2.889	3.197	2.989	3.302	3.451	3.477
NEUROPSYCHOLOGICAL REHABILITATION	11	2.7	2.928	2.868	2.556	2.667	2.842	2.28	2.082	1.955	2.068	2.011
NEUROPSYCHOLOGY	11	2.4	3.424	3.295	2.506	2.477	2.699	3.286	2.879	3.269	3.425	3.579
NEUROPSYCHOLOGY REVIEW	11	5.8	6.94	7.444	4.84	5.739	4.894	6.352	6.061	4.592	5.4	6.42
NEUROPSYCHOPHARMACOLOGY	11	7.6	8.304	7.855	6.751	7.16	6.544	6.403	6.399	7.048	7.833	8.678
NEUROREPORT	11	1.7	1.703	1.837	1.394	1.146	1.266	1.395	1.343	1.52	1.644	1.404
NEUROSCIENCE	11	3.3	3.708	3.59	3.056	3.244	3.382	3.277	3.231	3.357	3.327	3.122
NEUROSCIENCE AND BIOBEHAVIORAL REVIEWS	11	8.2	9.052	8.989	8.329	8.002	8.037	8.299	8.58	8.802	10.284	9.44
NEUROSCIENCE BULLETIN	11	5.6	5.271	5.203	4.326	4.246	3.155	2.624	2.322	2.509	1.832	1.365
NEUROSCIENCE LETTERS	11	2.5	3.197	3.046	2.274	2.173	2.159	2.18	2.107	2.03	2.055	2.026
NEUROSCIENCE RESEARCH	11	2.9	2.904	3.322	2.645	2.071	2.277	2.06	2.004	1.937	2.145	2.204
NEUROSCIENTIST	11	5.6	7.235	7.519	6.5	6.791	7.461	7.391	7.295	6.837	7.618	5.633
NEUROTHERAPEUTICS	11	5.7	6.088	7.62	6.035	5.552	5.719	5.166	4.676	5.054	3.883	5.904
NEUROTOXICITY RESEARCH	11	3.7	3.978	3.911	2.992	3.311	3.186	2.942	3.14	3.538	3.151	2.865
NEUROTOXICOLOGY	11	3.4	4.398	4.294	3.105	3.263	3.076	3.1	2.738	3.379	3.054	2.652
NEUROTOXICOLOGY AND TERATOLOGY	11	2.9	4.071	3.763	3.274	2.902	2.811	2.41	2.488	2.762	3.224	3.181
NUTRITIONAL NEUROSCIENCE	11	3.6	4.062	5	4.028	3.95	3.313	3.765	2.616	2.274	2.114	1.647
PAIN	11	7.4	7.926	6.961	5.483	6.029	5.559	5.445	5.557	5.213	5.836	5.644
PHARMACOLOGY BIOCHEMISTRY AND BEHAVIOR	11	3.6	3.697	3.533	2.519	2.773	2.538	2.748	2.537	2.781	2.82	2.608
PROGRESS IN NEUROBIOLOGY	11	6.7	10.885	11.685	9.371	10.658	14.163	13.217	13.177	9.992	10.301	9.035
PROGRESS IN NEURO-PSYCHOPHARMACOLOGY & BIOLOGICAL PSYCHIATRY	11	5.6	5.201	5.067	4.361	4.315	4.185	4.187	4.361	3.689	4.025	3.552
PSYCHIATRIC GENETICS	11	0.9	2.574	2.458	1.267	1.375	1.586	1.557	1.736	1.941	2.274	2.365

Journal Name	Active Years	2022 Impact Factor	2021 Impact Factor	2020 Impact Factor	2019 Impact Factor	2018 Impact Factor	2017 Impact Factor	2016 Impact Factor	2015 Impact Factor	2014 Impact Factor	2013 Impact Factor	2012 Impact Factor
PSYCHIATRY AND CLINICAL NEUROSCIENCES	11	11.9	12.145	5.188	3.351	3.489	3.199	2.063	2.025	1.634	1.62	2.04
PSYCHONEUROENDOCRINOLOGY	11	3.7	4.693	4.905	4.732	4.013	4.731	4.788	4.704	4.944	5.591	5.137
PSYCHOPHARMACOLOGY	11	3.4	4.415	4.53	3.13	3.424	3.222	3.308	3.54	3.875	3.988	4.061
PSYCHOPHYSIOLOGY	11	3.7	4.348	4.016	3.692	3.378	3.118	2.668	3.074	2.986	3.18	3.261
PURINERGIC SIGNALLING	11	3.5	3.95	3.765	3.065	3.038	3.19	3.022	3.196	3.886	3.51	2.635
RESTORATIVE NEUROLOGY AND NEUROSCIENCE	11	2.8	2.976	2.406	2.378	1.839	2.101	2.526	2.661	2.49	4.179	2.929
REVIEWS IN THE NEUROSCIENCES	11	4.1	4.703	4.353	3.358	2.157	2.59	2.546	3.198	3.33	3.314	3.26
SEIZURE-EUROPEAN JOURNAL OF EPILEPSY	11	3	3.414	3.184	2.522	2.765	2.839	2.448	2.109	1.822	2.059	2.004
SLEEP	11	5.6	6.313	5.849	4.805	4.571	5.135	4.923	4.793	4.591	5.062	5.1
SLEEP AND BIOLOGICAL RHYTHMS	11	1.1	1.39	1.186	0.925	0.752	0.655	0.926	0.628	0.588	0.759	1.052
SLEEP MEDICINE REVIEWS	11	10.5	11.401	11.609	9.607	10.517	10.602	8.958	7.341	8.513	9.141	8.681
SOCIAL COGNITIVE AND AFFECTIVE NEUROSCIENCE	11	4.2	4.235	3.436	3.571	3.662	3.5	3.937	5.101	7.372	5.884	5.042
SOCIAL NEUROSCIENCE	11	2	2.381	2.083	2.248	2.154	2.575	2.255	2.778	2.66	2.873	2.785
SOMATOSENSORY AND MOTOR RESEARCH	11	0.9	1.126	1.111	1.028	1.235	1.185	0.909	0.778	0.641	0.577	0.931
STEREOTACTIC AND FUNCTIONAL NEUROSURGERY	11	1.7	1.643	1.875	1.635	1.905	1.648	1.692	1.691	2.019	1.477	1.458
STRESS-THE INTERNATIONAL JOURNAL ON THE BIOLOGY OF STRESS	11	2.3	3.34	3.493	3.102	2.168	3.047	2.59	2.383	2.715	3.463	3.252
SYNAPSE	11	2.3	2.537	2.562	2.318	2.545	2.36	2.132	2.026	2.127	2.428	2.31
TRANSLATIONAL NEUROSCIENCE	11	2.1	1.264	1.757	1.373	2.038	0.833	0.922	1.012	1.319	0.716	0.482
TRENDS IN COGNITIVE SCIENCES	11	19.9	24.482	20.229	15.218	16.173	15.557	15.402	17.85	21.965	21.147	16.008
TRENDS IN NEUROSCIENCES	11	15.9	16.978	13.837	12.891	12.314	11.439	11.124	12.504	13.555	12.902	13.582
VISION RESEARCH	11	1.8	1.984	1.886	2.61	2.178	2.069	1.971	1.776	1.815	2.381	2.137
VISUAL NEUROSCIENCE	11	1.9	1.895	3.241	3	1.645	1.927	1.737	1.871	2.207	1.676	1.475
ZHURNAL VYSSHEI NERVNOI DEYATELNOSTI IMENI I P PAVLOVA	11	0.3	0.278	0.437	0.432	0.269	0.311	0.236	0.175	0.11	0.213	0.248

Growth Rate Journals 10 Years

ID	Journal Name	2022	2021	2020	2019	2018	2017	2016	2015	2014	2013	Slope	Slope sign
1	ACS CHEMICAL NEUROSCIENCE	-13.5	30.8	-1.5	16.2	-8.3	8.4	-10.7	-0.3	3.6	8.8	-0.292	0.0
2	ACTA NEUROBIOLOGIAE EXPERIMENTALIS	10.3	-19.6	2.5	0.8	1.9	24.3	-29.3	32.8	-42.7	13.5	-0.297	0.0
3	ACTA NEUROLOGICA BELGICA	9.3	3.1	20.5	23.4	-22.2	20.3	15.2	67.2	49.5	28.3	4.532	1.0
4	ACTA NEUROPATHOLOGICA	-20.1	-7.0	19.9	-21.6	14.5	30.0	7.5	5.6	10.1	0.4	2.033	1.0
5	ACTA NEUROPSYCHIATRICA	-15.8	32.6	13.4	51.7	-15.2	20.3	155.1	-5.2	25.5	5.4	2.388	1.0
6	ACTAS ESPANOLAS DE PSIQUIATRIA	-10.0	39.4	-28.9	13.7	68.5	-34.4	64.7	-32.3	58.1	67.9	5.247	1.0
7	ACUPUNCTURE & ELECTRO-THERAPEUTICS RESEARCH	-56.1	378.3	-28.5	-52.0	-4.1	-50.0	11.8	-5.6	106.0	-45.7	-9.407	0.0
8	ALZHEIMERS RESEARCH & THERAPY	1.9	26.5	14.2	-0.4	22.5	-18.5	18.4	30.6	13.7	-20.3	-1.16	0.0
9	ANNALS OF NEUROLOGY	-0.7	8.2	15.3	-4.8	-7.4	3.6	2.6	-3.4	-16.2	6.4	-1.015	0.0
10	ANNUAL REVIEW OF NEUROSCIENCE	-10.6	24.9	-0.6	4.0	-17.9	-6.1	9.6	-26.2	-14.7	9.9	-1.163	0.0
11	ARCHIVES ITALIENNES DE BIOLOGIE	-38.2	61.9	-15.2	21.0	63.7	2.6	-4.9	-59.0	4.6	-0.8	-2.556	0.0
12	ARQUIVOS DE NEURO-PSIQUIATRIA	-31.2	43.3	40.9	-3.8	3.3	12.5	-3.7	11.2	-16.2	21.6	-0.485	0.0
13	ASN NEURO	-9.6	25.4	-0.5	53.9	-25.2	19.4	7.1	-29.6	-9.4	21.9	-1.221	0.0
14	AUDIOLOGY AND NEURO-OTOLOGY	-27.7	19.4	19.7	-24.5	-1.2	16.0	0.8	4.2	-7.9	-20.1	-0.648	0.0
15	AUTONOMIC NEUROSCIENCE-BASIC & CLINICAL	14.6	-25.1	43.0	-2.1	-13.7	17.1	37.3	3.8	13.8	-25.7	-0.831	0.0
16	BEHAVIORAL AND BRAIN FUNCTIONS	29.1	5.1	76.9	-13.5	0.3	11.0	28.3	-12.8	-1.4	-28.3	-5.298	0.0
17	BEHAVIORAL AND BRAIN SCIENCES	37.2	69.8	-27.4	0.8	14.1	6.1	-30.4	-1.7	38.8	-19.4	-4.239	0.0
18	BEHAVIORAL NEUROSCIENCE	-11.8	12.7	11.6	-18.5	-16.2	2.2	-8.8	-1.4	-16.1	23.4	0.595	1.0
19	BEHAVIOURAL BRAIN RESEARCH	-19.5	0.6	11.9	7.5	-12.7	5.7	0.0	-0.9	-10.7	1.9	0.275	1.0
20	BEHAVIOURAL PHARMACOLOGY	-29.7	-0.7	31.7	-2.6	-3.6	-16.4	10.9	-6.9	-2.1	-4.7	0.307	1.0
21	BIOLOGICAL CYBERNETICS	-38.2	47.3	87.8	-14.9	-9.4	-16.1	6.5	-6.0	-11.4	-6.5	-3.252	0.0
22	BIOLOGICAL PSYCHIATRY	-17.3	-4.3	10.6	5.2	-4.0	5.0	1.8	9.3	8.3	2.4	1.559	1.0
23	BIPOLAR DISORDERS	10.4	-20.7	24.7	9.6	9.9	-0.9	-7.2	-1.7	1.6	5.8	-0.473	0.0
24	BMC NEUROSCIENCE	-26.5	-0.7	17.0	7.3	20.6	-6.0	0.3	-13.5	-6.3	-5.2	-0.287	0.0
25	BRAIN	-4.9	13.0	19.1	-4.0	8.9	5.4	1.9	9.9	-10.1	3.1	-0.731	0.0

ID	Journal Name	2022	2021	2020	2019	2018	2017	2016	2015	2014	2013	Slope	Slope sign
26	BRAIN AND COGNITION	-6.8	16.1	-7.9	-4.2	1.7	5.8	1.4	-3.1	-7.7	-5.0	-0.639	0.0
27	BRAIN AND LANGUAGE	-10.1	16.8	1.8	-13.4	-5.3	16.9	-19.7	-5.5	-2.8	-2.3	-0.608	0.0
28	BRAIN BEHAVIOR AND EVOLUTION	-11.4	6.1	-3.6	21.6	-6.5	-13.8	-8.9	4.5	-53.1	48.6	0.408	1.0
29	BRAIN BEHAVIOR AND IMMUNITY	-21.5	166.4	8.8	7.5	-2.2	5.7	1.5	-0.3	-3.9	9.2	-5.888	0.0
30	BRAIN IMPAIRMENT	-29.8	-34.0	27.4	41.5	-1.9	62.8	6.2	-30.2	-7.9	35.1	2.655	1.0
31	BRAIN INJURY	-12.3	-6.2	36.7	1.5	-19.2	4.6	8.2	0.8	-2.8	23.0	1.246	1.0
32	BRAIN PATHOLOGY	-15.9	16.9	16.9	-12.3	2.7	-6.6	26.0	13.2	6.6	-8.1	0.517	1.0
33	BRAIN RESEARCH	-19.7	11.0	19.0	-6.7	-6.3	13.8	7.2	-9.9	0.5	-1.8	0.03	1.0
34	BRAIN RESEARCH BULLETIN	2.3	-8.9	21.0	8.6	-9.8	13.4	17.9	-5.4	-8.6	1.3	-0.529	0.0
35	BRAIN STIMULATION	-16.2	2.6	36.4	-5.1	13.1	0.7	26.8	9.0	-19.0	19.7	0.714	1.0
36	BRAIN STRUCTURE & FUNCTION	-17.3	14.6	-0.8	-8.9	-14.4	-9.9	-19.2	3.4	23.0	-41.7	-1.005	0.0
37	BRAIN TOPOGRAPHY	-36.8	41.6	9.5	-11.1	14.8	-20.4	-8.9	7.5	37.7	-31.4	-0.101	0.0
38	CELLULAR AND MOLECULAR NEUROBIOLOGY	-5.5	-16.2	39.9	-5.4	-2.2	32.5	26.2	-7.1	13.9	-4.0	0.712	1.0
39	CEPHALALGIA	-19.3	-3.5	29.3	9.7	14.2	7.7	-40.4	23.7	18.7	18.2	1.873	1.0
40	CEREBELLUM	-4.1	-5.2	22.9	-8.3	6.7	-1.1	33.1	-10.6	-5.1	10.4	0.479	1.0
41	CEREBRAL CORTEX	-23.9	-9.3	6.2	-7.2	-13.8	-3.8	-20.8	-4.4	4.3	21.6	2.551	1.0
42	CESKA A SLOVENSKA NEUROLOGIE A NEUROCHIRURGIE	21.7	17.4	-7.2	6.2	-30.1	38.0	76.1	26.7	3.8	-57.3	-2.175	0.0
43	CHEMICAL SENSES	-15.7	57.8	39.8	-3.2	-27.8	28.4	0.8	-20.8	-3.7	1.7	-3.075	0.0
44	CHEMOSENSORY PERCEPTION	-24.4	-27.8	-8.4	142.7	-49.8	11.3	40.0	-19.1	-4.6	3.6	0.691	1.0
45	CLINICAL AUTONOMIC RESEARCH	3.1	26.8	49.4	19.4	52.0	28.1	1.5	-15.5	-20.2	26.1	-3.179	0.0
46	CLINICAL EEG AND NEUROSCIENCE	-2.2	11.0	4.4	-3.1	0.8	-16.5	16.2	-16.1	-29.7	73.7	2.037	1.0
47	CLINICAL NEUROPHYSIOLOGY	-3.3	31.1	15.4	-12.5	1.7	-6.5	12.8	10.6	4.0	-5.2	-0.989	0.0
48	CNS NEUROSCIENCE & THERAPEUTICS	-21.8	34.2	28.7	20.0	-2.9	-10.8	-2.5	2.2	3.9	-15.1	-2.179	0.0
49	COGNITIVE AFFECTIVE & BEHAVIORAL NEUROSCIENCE	-17.8	7.4	48.8	-17.1	3.7	-21.4	13.1	-12.2	2.4	-17.0	-1.623	0.0
50	COGNITIVE COMPUTATION	10.4	-9.7	25.8	0.5	23.2	1.1	78.0	34.2	30.9	26.9	4.153	1.0
51	COGNITIVE NEURODYNAMICS	6.5	-31.7	29.5	29.9	51.1	9.4	-15.3	29.2	-5.6	1.6	-0.246	0.0

ID	Journal Name	2022	2021	2020	2019	2018	2017	2016	2015	2014	2013	Slope	Slope sign
52	COGNITIVE NEUROSCIENCE	-21.6	-16.8	2.2	-10.7	-1.6	82.7	-21.2	-10.6	11.7	8.3	2.777	1.0
53	COGNITIVE SYSTEMS RESEARCH	-14.1	28.9	85.2	37.4	-2.9	20.6	-1.8	44.9	10.2	0.5	-1.788	0.0
54	CORTEX	-22.5	15.3	0.4	-6.2	-12.9	14.7	-0.8	-15.9	-15.1	-1.9	-0.4	0.0
55	CURRENT ALZHEIMER RESEARCH	-30.9	-13.1	14.8	-6.8	-0.5	11.4	-6.1	-19.1	2.4	3.3	1.581	1.0
56	CURRENT NEUROLOGY AND NEUROSCIENCE REPORTS	-7.1	18.7	16.1	28.7	-2.2	4.0	13.0	-3.2	-16.6	-3.0	-2.107	0.0
57	CURRENT NEUROPHARMACOLOGY	-31.2	4.3	58.3	2.2	12.3	20.9	-10.3	23.1	29.9	15.6	2.396	1.0
58	CURRENT NEUROVASCULAR RESEARCH	-8.5	15.3	20.7	-8.9	5.5	-25.3	8.2	-5.8	-17.6	-3.8	-1.82	0.0
59	CURRENT OPINION IN NEUROBIOLOGY	-19.4	6.7	5.8	4.1	-8.1	6.7	-3.8	-3.8	-2.0	-7.8	-0.084	0.0
60	CURRENT OPINION IN NEUROLOGY	-23.6	10.0	35.7	-9.5	15.9	-14.7	5.1	-15.8	-7.4	5.8	-0.616	0.0
61	DEVELOPMENTAL COGNITIVE NEUROSCIENCE	-19.1	-10.1	30.2	0.9	2.2	11.4	9.0	3.4	3.5	17.2	1.951	1.0
62	DEVELOPMENTAL NEUROBIOLOGY	-3.3	-21.7	0.7	51.3	0.1	-12.6	17.5	-25.0	-19.6	-5.3	-1.486	0.0
63	DEVELOPMENTAL NEUROSCIENCE	-15.2	14.6	-1.9	43.1	-0.4	-29.7	4.7	7.5	9.9	-28.1	-1.497	0.0
64	ENCEPHALE-REVUE DE PSYCHIATRIE CLINIQUE BIOLOGIQUE ET THERAPEUTIQUE	-3.1	115.9	47.9	0.9	44.4	-19.3	9.9	-3.3	16.7	21.3	-4.648	0.0
65	EUROPEAN JOURNAL OF NEUROLOGY	-18.9	3.3	34.8	2.9	-5.1	15.9	0.8	-2.4	5.3	-7.4	-0.332	0.0
66	EUROPEAN JOURNAL OF NEUROSCIENCE	-8.1	9.2	8.7	11.9	-1.7	-3.7	-1.1	-6.5	-13.3	-2.2	-1.347	0.0
67	EUROPEAN JOURNAL OF PAIN	-1.4	-7.2	12.7	9.5	6.6	-0.9	4.1	-1.0	-9.0	4.9	-0.289	0.0
68	EUROPEAN NEUROLOGY	4.7	34.0	44.7	-4.3	-20.9	-8.0	21.0	3.5	-0.4	-9.2	-2.932	0.0
69	EUROPEAN NEUROPSYCHOPHARMACOLOGY	3.4	17.7	19.4	-13.8	8.2	-2.6	-3.9	0.9	-19.0	17.4	-1.24	0.0
70	EXPERIMENTAL BRAIN RESEARCH	-3.1	4.7	23.9	-15.3	4.0	-5.8	-6.8	1.0	-6.1	-2.4	-1.017	0.0
71	EXPERIMENTAL NEUROLOGY	-5.7	5.4	13.6	2.8	1.8	-4.7	1.1	-0.8	1.7	-0.6	-0.39	0.0
72	FOLIA NEUROPATHOLOGICA	-19.8	22.4	59.5	10.2	-13.8	23.1	-11.4	-21.4	-5.9	7.8	-2.315	0.0
73	FRONTIERS IN AGING NEUROSCIENCE	-15.8	-0.8	31.8	20.1	1.4	-20.5	3.6	8.7	40.7	-45.6	-0.993	0.0
74	FRONTIERS IN BEHAVIORAL NEUROSCIENCE	-17.1	1.7	41.6	-4.2	-16.4	1.1	-8.5	3.7	-21.4	-12.6	-1.854	0.0
75	FRONTIERS IN CELLULAR NEUROSCIENCE	-13.8	11.7	40.4	0.5	-9.3	-5.6	-1.2	7.5	2.7	-6.6	-0.993	0.0
76	FRONTIERS IN COMPUTATIONAL NEUROSCIENCE	-5.5	42.3	-6.1	9.1	12.1	13.8	-31.4	20.5	-1.4	-10.0	-2.018	0.0
77	FRONTIERS IN HUMAN NEUROSCIENCE	-16.5	9.6	18.6	-6.9	0.0	-10.5	-11.7	0.2	25.3	-0.4	0.836	1.0

ID	Journal Name	2022	2021	2020	2019	2018	2017	2016	2015	2014	2013	Slope	Slope sign
78	FRONTIERS IN NEURAL CIRCUITS	4.7	-4.3	10.6	1.8	-1.0	4.2	-22.5	8.7	20.9	-11.5	-0.283	0.0
79	FRONTIERS IN NEUROANATOMY	-18.1	-8.1	17.1	12.6	-7.3	-3.5	0.2	-8.0	-15.1	2.9	-0.114	0.0
80	FRONTIERS IN NEUROENDOCRINOLOGY	-11.2	-3.2	-4.8	15.2	14.2	-27.1	6.5	25.8	-7.2	-5.1	0.685	1.0
81	GAIT & POSTURE	-12.6	-3.3	20.9	-2.7	6.2	-3.2	2.7	-16.9	19.7	16.8	1.472	1.0
82	GENES BRAIN AND BEHAVIOR	-32.6	7.5	1.5	7.6	-9.7	-6.6	14.3	-10.5	4.5	-2.6	1.282	1.0
83	GLIA	-23.2	8.3	24.5	2.7	-0.3	-5.7	3.4	-0.6	10.3	7.9	1.001	1.0
84	HEARING RESEARCH	-23.7	14.5	-13.1	25.1	4.5	-2.8	-18.5	20.1	4.2	12.3	1.7	1.0
85	HIPPOCAMPUS	-6.7	-3.7	14.5	4.2	-17.6	0.5	-3.2	-2.1	-3.3	-21.7	-1.322	0.0
86	HUMAN BRAIN MAPPING	-11.1	7.2	14.0	-2.9	-7.6	8.8	-8.7	-16.9	-13.8	0.7	-1.188	0.0
87	HUMAN MOVEMENT SCIENCE	-12.4	10.9	3.1	8.7	4.8	-0.1	14.6	0.5	-21.2	-1.8	-0.784	0.0
88	IDEGGYOGYASZATI SZEMLE-CLINICAL NEUROSCIENCE	13.0	65.8	26.7	198.2	-55.2	-21.7	-14.4	-2.6	12.5	-1.4	-7.598	0.0
89	INTERNATIONAL JOURNAL OF DEVELOPMENTAL NEUROSCIENCE	-29.1	3.4	28.6	-19.3	-5.1	21.9	-14.0	-7.8	-11.6	8.4	0.571	1.0
90	INTERNATIONAL JOURNAL OF NEUROPSYCHOPHARMACOLOGY	-15.5	9.7	19.5	3.0	5.7	-15.5	8.7	8.1	-23.8	-6.7	-1.312	0.0
91	INTERNATIONAL JOURNAL OF NEUROSCIENCE	-15.1	13.0	8.8	13.8	0.2	5.6	1.9	12.9	-0.5	25.7	1.591	1.0
92	INTERNATIONAL JOURNAL OF PSYCHOPHYSIOLOGY	3.3	-3.1	13.9	9.3	-16.1	11.1	-0.5	-9.9	8.8	30.1	1.229	1.0
93	JARO-JOURNAL OF THE ASSOCIATION FOR RESEARCH IN OTOLARYNGOLOGY	-26.4	6.7	10.8	1.6	0.0	10.6	-19.0	16.6	2.0	-13.7	0.361	1.0
94	JOURNAL OF ALZHEIMERS DISEASE	-3.8	-7.0	14.4	11.1	1.2	-6.8	-4.8	-5.6	14.9	-13.5	-0.54	0.0
95	JOURNAL OF CEREBRAL BLOOD FLOW AND METABOLISM	-4.5	6.4	9.1	-5.9	-0.1	19.0	3.1	-8.8	1.3	-1.1	-0.297	0.0
96	JOURNAL OF CHEMICAL NEUROANATOMY	-9.6	1.5	29.7	-0.2	9.0	12.3	6.9	20.0	-40.5	1.8	-1.302	0.0
97	JOURNAL OF CLINICAL NEUROPHYSIOLOGY	-7.3	19.0	51.8	-14.3	-15.6	61.9	-8.5	-6.4	-10.4	10.2	-1.478	0.0
98	JOURNAL OF CLINICAL NEUROSCIENCE	-5.5	7.9	11.4	10.5	-2.9	5.3	12.3	0.7	4.6	5.2	0.195	1.0
99	JOURNAL OF COGNITIVE NEUROSCIENCE	-6.4	6.0	3.9	2.5	-12.7	11.6	-12.7	-12.9	-12.8	4.3	-0.851	0.0
100	JOURNAL OF COMPARATIVE NEUROLOGY	-17.4	-5.8	14.8	-13.5	-4.7	4.1	-2.0	3.3	-8.1	-4.2	0.543	1.0

ID	Journal Name	2022	2021	2020	2019	2018	2017	2016	2015	2014	2013	Slope	Slope sign
101	JOURNAL OF COMPARATIVE PHYSIOLOGY A-NEUROETHOLOGY SENSORY NEURAL AND BEHAVIORAL PHYSIOLOGY	-12.1	30.1	21.1	-19.4	-4.5	-18.9	22.2	-2.4	24.6	-12.0	-0.268	0.0
102	JOURNAL OF COMPUTATIONAL NEUROSCIENCE	-17.4	-10.4	-10.5	15.5	-2.4	8.3	-20.7	7.6	-16.7	-14.4	-0.151	0.0
103	JOURNAL OF ELECTROMYOGRAPHY AND KINESIOLOGY	-5.3	11.5	36.1	-0.7	11.8	3.8	-1.3	-7.1	-4.5	4.9	-1.488	0.0
104	JOURNAL OF HEADACHE AND PAIN	-13.8	18.0	51.7	22.4	15.1	-4.9	2.4	24.8	-14.6	18.1	-0.945	0.0
105	JOURNAL OF INTEGRATIVE NEUROSCIENCE	8.2	-21.4	77.5	4.7	72.1	2.3	-18.2	-15.4	-16.6	-2.2	-4.014	0.0
106	JOURNAL OF MOLECULAR NEUROSCIENCE	8.2	-16.8	28.6	3.9	5.0	10.1	-5.2	0.4	-15.0	-4.6	-1.614	0.0
107	JOURNAL OF MOTOR BEHAVIOR	3.1	2.3	3.8	-2.6	-13.2	14.0	-15.6	10.9	0.9	34.9	1.82	1.0
108	JOURNAL OF MUSCULOSKELETAL & NEURONAL INTERACTIONS	1.9	-8.7	23.0	6.3	-5.4	10.9	-9.1	-6.1	-27.3	-2.2	-2.075	0.0
109	JOURNAL OF NEURAL ENGINEERING	-20.7	-6.2	29.9	-9.0	16.1	13.1	-0.8	6.0	-3.5	4.1	0.872	1.0
110	JOURNAL OF NEURAL TRANSMISSION	-14.3	7.7	2.0	20.7	4.5	16.2	-7.5	7.7	-16.3	-5.9	-0.834	0.0
111	JOURNAL OF NEUROCHEMISTRY	-15.3	3.2	32.1	-16.5	5.7	12.9	6.3	-10.3	0.9	6.8	0.278	1.0
112	JOURNAL OF NEURODEVELOPMENTAL DISORDERS	20.3	1.2	15.4	-2.9	2.6	-2.3	28.5	-14.7	-11.8	7.5	-1.624	0.0
113	JOURNAL OF NEUROENDOCRINOLOGY	-17.3	6.7	25.7	-5.1	-10.4	-2.2	9.4	1.1	-10.5	5.3	0.069	1.0
114	JOURNAL OF NEUROENGINEERING AND REHABILITATION	-2.1	22.2	21.1	-1.8	-7.3	9.9	45.3	-11.7	4.5	2.1	-0.555	0.0
115	JOURNAL OF NEUROGENETICS	12.0	35.7	-13.1	-15.3	10.5	-33.0	23.6	46.2	-8.3	-35.9	-2.243	0.0
116	JOURNAL OF NEUROIMMUNE PHARMACOLOGY	-14.9	75.7	0.8	6.3	5.6	9.7	-14.3	-5.2	29.6	-16.6	-2.579	0.0
117	JOURNAL OF NEUROIMMUNOLOGY	2.5	-7.4	11.3	10.3	6.7	-2.4	7.3	2.8	-11.5	-8.1	-1.119	0.0
118	JOURNAL OF NEUROINFLAMMATION	-3.0	15.2	43.7	1.6	9.8	1.8	9.3	-13.7	10.3	12.7	-1	0.0
119	JOURNAL OF NEUROLINGUISTICS	-15.7	38.8	-7.7	48.6	-19.4	10.3	8.3	-13.0	-6.7	43.1	0.568	1.0
120	JOURNAL OF NEUROPATHOLOGY AND EXPERIMENTAL NEUROLOGY	1.7	-14.6	26.1	-15.5	-0.9	-0.4	2.1	-9.6	-13.2	0.6	-0.754	0.0
121	JOURNAL OF NEUROPHYSIOLOGY	-15.9	9.6	21.5	-14.5	4.5	4.4	-9.7	-8.1	-5.1	-7.9	-0.99	0.0
122	JOURNAL OF NEUROPSYCHIATRY AND CLINICAL NEUROSCIENCES	0.3	31.5	0.3	11.2	6.3	0.4	-24.1	-13.6	1.9	15.4	-1.537	0.0

ID	Journal Name	2022	2021	2020	2019	2018	2017	2016	2015	2014	2013	Slope	Slope sign
123	JOURNAL OF NEUROSCIENCE	-21.0	8.8	8.7	-6.6	1.7	-0.3	1.1	-6.6	-6.0	-2.3	0.055	1.0
124	JOURNAL OF NEUROSCIENCE METHODS	0.4	25.0	7.9	-20.5	4.4	4.5	24.4	1.4	3.4	-7.3	-0.723	0.0
125	JOURNAL OF NEUROSCIENCE RESEARCH	-5.3	6.5	-11.4	13.5	55.5	7.3	-7.7	3.7	-4.9	-8.2	-0.869	0.0
126	JOURNAL OF NEUROTRAUMA	-13.7	-7.6	29.9	8.0	-25.0	-3.6	18.6	17.9	-6.4	-7.6	0.34	1.0
127	JOURNAL OF NEUROVIROLOGY	-14.4	41.5	12.3	2.3	-28.7	0.7	24.8	-1.0	-21.9	16.6	-0.812	0.0
128	JOURNAL OF PAIN	-25.7	-7.6	26.1	-14.8	11.6	7.5	1.3	11.3	-4.9	30.1	2.979	1.0
129	JOURNAL OF PARKINSONS DISEASE	-5.8	-0.9	7.5	40.0	16.6	25.0	-15.8	57.9	74.1	1.3	4.128	1.0
130	JOURNAL OF PHYSIOLOGY-LONDON	-11.7	20.2	14.0	-8.8	9.8	-4.2	0.2	-6.1	10.8	3.7	-0.084	0.0
131	JOURNAL OF PINEAL RESEARCH	-14.7	-7.1	-10.5	-4.6	31.1	11.8	11.6	-3.0	22.9	7.0	2.859	1.0
132	JOURNAL OF PSYCHIATRY & NEUROSCIENCE	-24.5	-7.9	41.2	-10.6	-8.7	3.9	-7.3	-5.0	-21.8	20.0	0.58	1.0
133	JOURNAL OF PSYCHOPHARMACOLOGY	-10.1	9.8	33.1	-26.1	-10.9	13.4	14.9	-6.7	14.8	0.7	0.484	1.0
134	JOURNAL OF PSYCHOPHYSIOLOGY	5.8	-7.8	-5.2	40.6	9.1	34.3	-41.5	-26.6	11.6	42.5	0.837	1.0
135	JOURNAL OF SLEEP RESEARCH	-16.9	33.0	9.9	5.6	0.0	5.3	5.4	-7.6	13.5	-3.1	-0.575	0.0
136	JOURNAL OF STROKE & CEREBROVASCULAR DISEASES	-6.6	25.3	19.5	8.6	3.0	5.3	-5.1	-4.2	-16.3	0.5	-2.333	0.0
137	JOURNAL OF THE HISTORY OF THE NEUROSCI-ENCES	-22.4	21.7	52.4	42.2	-15.3	-54.5	19.7	-5.9	4.7	90.4	3.012	1.0
138	JOURNAL OF THE INTERNATIONAL NEUROPSY-CHOLOGICAL SOCIETY	-16.5	7.7	12.3	-16.8	11.6	27.3	-17.2	-11.1	-1.5	11.6	0.521	1.0
139	JOURNAL OF THE NEUROLOGICAL SCIENCES	-3.4	43.1	2.1	17.5	8.3	6.7	7.9	-14.1	9.4	0.8	-1.877	0.0
140	JOURNAL OF THE PERIPHERAL NERVOUS SYS-TEM	-26.8	48.5	41.7	1.0	-4.3	8.0	4.6	-18.1	10.1	-2.6	-1.981	0.0
141	JOURNAL OF VESTIBULAR RESEARCH-EQUILIBRIUM & ORIENTATION	-2.3	-3.3	-13.5	1.5	-3.2	218.3	-14.0	-12.0	-18.3	45.6	3.084	1.0
142	LEARNING & MEMORY	-25.9	9.7	4.3	-0.6	-11.2	-7.7	-0.4	-20.5	-16.4	7.8	0.004	1.0
143	METABOLIC BRAIN DISEASE	-1.5	2.0	31.5	13.1	-1.2	6.3	-11.8	-1.3	10.0	2.8	-0.825	0.0
144	MOLECULAR AND CELLULAR NEUROSCIENCE	-24.3	7.2	35.6	11.5	-13.8	7.4	-14.3	-6.3	2.8	-2.7	-0.614	0.0
145	MOLECULAR BRAIN	-18.2	8.9	-13.8	15.7	17.5	1.1	-8.9	-23.6	12.8	3.4	0.5	1.0
146	MOLECULAR NEUROBIOLOGY	-10.3	1.7	24.2	-1.9	-9.7	-18.0	14.7	5.1	-2.8	-3.4	-0.145	0.0
147	MOLECULAR NEURODEGENERATION	-20.1	33.1	47.9	16.0	28.8	-5.2	4.1	-0.8	24.2	31.9	0.56	1.0

ID	Journal Name	2022	2021	2020	2019	2018	2017	2016	2015	2014	2013	Slope	Slope sign
148	MOLECULAR PAIN	-2.1	-0.7	25.9	-1.8	-14.3	-9.3	15.1	-16.0	3.5	-6.4	-0.991	0.0
149	MOLECULAR PSYCHIATRY	-18.1	-16.0	29.1	3.4	2.9	-11.8	-0.8	-8.2	-4.3	1.7	0.28	1.0
150	MOTOR CONTROL	-28.3	7.9	39.8	-21.9	36.1	27.6	-27.7	-15.9	-15.1	4.5	-1.031	0.0
151	MUSCLE & NERVE	-11.7	19.7	28.4	4.7	-4.1	-4.2	-4.0	18.8	-1.2	-0.1	-0.704	0.0
152	NATURE NEUROSCIENCE	-13.1	15.6	24.0	-5.0	6.1	11.6	6.7	3.9	7.5	-1.8	-0.092	0.0
153	NATURE REVIEWS NEUROSCIENCE	-10.5	11.1	3.6	1.5	1.6	13.0	-1.4	-6.8	0.2	-0.9	-0.245	0.0
154	NETWORK-COMPUTATION IN NEURAL SYSTEMS	420.0	17.8	154.6	-50.0	41.6	25.6	-13.1	-25.6	74.0	50.2	-22.679	0.0
155	NEURAL COMPUTATION	-11.5	61.8	-19.1	10.8	36.9	-14.8	19.2	-26.3	30.3	-3.8	-1.292	0.0
156	NEURAL DEVELOPMENT	-5.3	-1.1	46.1	13.5	8.8	2.6	-31.6	-12.0	2.4	-5.0	-2.456	0.0
157	NEURAL NETWORKS	-19.2	20.0	45.4	-4.3	-19.6	36.1	64.4	18.8	30.4	7.7	2.694	1.0
158	NEURAL PLASTICITY	-1.4	-12.6	16.4	-13.9	13.6	3.5	-14.4	-0.4	-0.7	26.0	1.42	1.0
159	NEURAL REGENERATION RESEARCH	0.7	18.0	61.9	28.3	10.7	26.3	82.7	340.0	-6.0	62.5	11.866	1.0
160	NEUROBIOLOGY OF AGING	-18.2	9.8	7.5	-1.2	-1.3	-13.0	-0.7	2.8	3.3	-21.3	-0.653	0.0
161	NEUROBIOLOGY OF DISEASE	-13.4	17.5	12.5	3.3	-1.3	4.1	3.4	-4.4	-2.4	-7.5	-0.997	0.0
162	NEUROBIOLOGY OF LEARNING AND MEMORY	-13.2	8.1	3.9	-8.0	-7.2	-8.4	3.0	-5.8	-9.5	21.3	1.031	1.0
163	NEUROCHEMICAL JOURNAL	11.6	-6.7	15.9	38.9	-26.2	18.8	17.2	-4.3	57.0	-17.9	0.359	1.0
164	NEUROCHEMICAL RESEARCH	-0.3	10.5	31.5	9.2	0.4	7.4	4.4	-4.7	1.6	20.0	-0.405	0.0
165	NEUROCHEMISTRY INTERNATIONAL	-2.3	9.6	1.0	-2.8	10.9	10.5	-3.6	9.5	16.7	-0.3	0.644	1.0
166	NEUROCIRUGIA	-2.1	47.7	-7.4	15.0	67.4	-43.4	33.7	39.9	-9.0	-6.1	-1.528	0.0
167	NEURODEGENERATIVE DISEASES	-12.2	14.8	23.1	-13.6	0.5	-2.0	-3.2	-16.3	1.7	1.3	-0.844	0.0
168	NEUROENDOCRINOLOGY	-20.2	4.5	15.1	-37.2	35.4	39.2	39.7	-40.9	-11.4	39.5	2.306	1.0
169	NEUROENDOCRINOLOGY LETTERS	9.7	-16.6	2.0	7.4	-7.4	-17.9	-3.0	18.4	-14.5	0.3	-0.181	0.0
170	NEUROGASTROENTEROLOGY AND MOTILITY	-11.6	10.1	22.1	-22.5	-1.0	6.2	9.3	-7.7	4.8	16.7	1.035	1.0
171	NEUROIMAGE	-23.0	12.9	11.1	1.5	7.1	-7.0	6.8	-14.1	3.7	-1.9	0.006	1.0
172	NEUROIMAGING CLINICS OF NORTH AMERICA	-12.3	15.9	-14.0	28.6	60.5	-3.8	-14.9	2.0	18.5	7.1	0.47	1.0
173	NEUROIMMUNOMODULATION	-14.1	12.2	47.9	24.7	-39.6	-16.3	13.3	25.5	5.8	-3.1	-0.413	0.0
174	NEUROINFORMATICS	4.7	-29.9	23.8	-35.6	33.1	20.4	11.7	1.4	-8.9	-1.1	0.676	1.0

ID	Journal Name	2022	2021	2020	2019	2018	2017	2016	2015	2014	2013	Slope	Slope sign
175	NEUROLOGIC CLINICS	-36.6	-0.5	30.8	3.9	-8.8	16.0	34.2	41.4	-13.2	19.7	3.561	1.0
176	NEUROLOGICAL RESEARCH	-24.9	3.2	2.1	21.1	36.9	5.3	-3.0	-1.5	-0.7	22.6	1.689	1.0
177	NEUROLOGICAL SCIENCES	-13.8	15.8	36.9	-2.8	8.7	30.6	-1.9	23.2	-3.2	5.9	0.001	1.0
178	NEUROLOGY INDIA	62.4	-21.4	-0.5	-21.4	25.0	23.2	24.7	14.4	13.7	3.8	-0.423	0.0
179	NEUROMOLECULAR MEDICINE	-14.7	6.8	46.2	2.1	-12.7	-10.2	-11.0	0.4	-5.3	-13.5	-2.058	0.0
180	NEUROMUSCULAR DISORDERS	-20.9	-17.6	37.9	19.3	5.0	-16.2	-4.4	17.8	-15.8	-9.5	-0.475	0.0
181	NEURON	-13.3	8.8	19.1	0.1	0.6	2.1	0.4	-7.2	-5.8	1.4	-0.603	0.0
182	NEUROPATHOLOGY	10.8	8.9	8.4	-18.6	14.5	5.8	14.7	-5.8	-8.1	-5.9	-1.509	0.0
183	NEUROPATHOLOGY AND APPLIED NEUROBIOLOGY	-20.0	-22.7	7.9	9.0	13.5	13.3	19.3	14.2	-21.0	2.7	1.691	1.0
184	NEUROPEPTIDES	-8.0	-4.1	36.3	0.2	-17.4	17.3	-8.8	3.1	3.8	23.2	1.078	1.0
185	NEUROPHARMACOLOGY	-10.9	0.4	18.5	1.5	2.8	-15.2	1.5	-3.3	6.0	17.1	0.993	1.0
186	NEUROPHYSIOLOGIE CLINIQUE-CLINICAL NEUROPHYSIOLOGY	-3.2	-17.0	46.3	17.8	6.0	28.4	7.7	19.5	-15.4	-42.7	-2.945	0.0
187	NEUROPHYSIOLOGY	-38.5	38.5	82.3	20.6	-27.4	77.8	3.5	2.6	12.1	-54.7	-4.094	0.0
188	NEUROPSYCHOBIOLOGY	-74.0	429.6	37.4	1.1	17.9	-4.7	-15.4	-22.0	-1.8	-2.9	-16.66	0.0
189	NEUROPSYCHOLOGIA	-14.9	-2.7	18.4	-7.7	-0.6	-9.6	7.0	-9.5	-4.3	-0.7	0.069	1.0
190	NEUROPSYCHOLOGICAL REHABILITATION	-7.8	2.1	12.2	-4.2	-6.2	24.6	9.5	6.5	-5.5	2.8	0.521	1.0
191	NEUROPSYCHOLOGY	-29.9	3.9	31.5	1.2	-8.2	-17.9	14.1	-11.9	-4.6	-4.3	-0.101	0.0
192	NEUROPSYCHOLOGY REVIEW	-16.4	-6.8	53.8	-15.7	17.3	-23.0	4.8	32.0	-15.0	-15.9	-0.851	0.0
193	NEUROPSYCHOPHARMACOLOGY	-8.5	5.7	16.4	-5.7	9.4	2.2	0.1	-9.2	-10.0	-9.7	-1.45	0.0
194	NEUROREPORT	-0.2	-7.3	31.8	21.6	-9.5	-9.2	3.9	-11.6	-7.5	17.1	-0.706	0.0
195	NEUROSCIENCE	-11.0	3.3	17.5	-5.8	-4.1	3.2	1.4	-3.8	0.9	6.6	0.389	1.0
196	NEUROSCIENCE AND BIOBEHAVIORAL REVIEWS	-9.4	0.7	7.9	4.1	-0.4	-3.2	-3.3	-2.5	-14.4	8.9	-0.107	0.0
197	NEUROSCIENCE BULLETIN	6.2	1.3	20.3	1.9	34.6	20.2	13.0	-7.5	37.0	34.2	2.313	1.0
198	NEUROSCIENCE LETTERS	-21.8	5.0	33.9	4.6	0.6	-1.0	3.5	3.8	-1.2	1.4	0.06	1.0
199	NEUROSCIENCE RESEARCH	-0.1	-12.6	25.6	27.7	-9.0	10.5	2.8	3.5	-9.7	-2.7	-1.021	0.0
200	NEUROSCIENTIST	-22.6	-3.8	15.7	-4.3	-9.0	0.9	1.3	6.7	-10.3	35.2	2.77	1.0

ID	Journal Name	2022	2021	2020	2019	2018	2017	2016	2015	2014	2013	Slope	Slope sign
201	NEUROTHERAPEUTICS	-6.4	-20.1	26.3	8.7	-2.9	10.7	10.5	-7.5	30.2	-34.2	-0.295	0.0
202	NEUROTOXICITY RESEARCH	-7.0	1.7	30.7	-9.6	3.9	8.3	-6.3	-11.2	12.3	10.0	0.189	1.0
203	NEUROTOXICOLOGY	-22.7	2.4	38.3	-4.8	6.1	-0.8	13.2	-19.0	10.6	15.2	0.965	1.0
204	NEUROTOXICOLOGY AND TERATOLOGY	-28.8	8.2	14.9	12.8	3.2	16.6	-3.1	-9.9	-14.3	1.4	-0.275	0.0
205	NUTRITIONAL NEUROSCIENCE	-11.4	-18.8	24.1	2.0	19.2	-12.0	43.9	15.0	7.6	28.4	3.582	1.0
206	PAIN	-6.6	13.9	27.0	-9.1	8.5	2.1	-2.0	6.6	-10.7	3.4	-1.021	0.0
207	PHARMACOLOGY BIOCHEMISTRY AND BEHAVIOR	-2.6	4.6	40.3	-9.2	9.3	-7.6	8.3	-8.8	-1.4	8.1	-0.939	0.0
208	PROGRESS IN NEUROBIOLOGY	-38.4	-6.8	24.7	-12.1	-24.7	7.2	0.3	31.9	-3.0	14.0	3.661	1.0
209	PROGRESS IN NEURO-PSYCHOPHARMACOLOGY & BIOLOGICAL PSYCHIATRY	7.7	2.6	16.2	1.1	3.1	0.0	-4.0	18.2	-8.3	13.3	-0.208	0.0
210	PSYCHIATRIC GENETICS	-65.0	4.7	94.0	-7.9	-13.3	1.9	-10.3	-10.6	-14.6	-3.8	-0.605	0.0
211	PSYCHIATRY AND CLINICAL NEUROSCIENCES	-2.0	134.1	54.8	-4.0	9.1	55.1	1.9	23.9	0.9	-20.6	-7.217	0.0
212	PSYCHONEUROENDOCRINOLOGY	-21.2	-4.3	3.7	17.9	-15.2	-1.2	1.8	-4.9	-11.6	8.8	0.862	1.0
213	PSYCHOPHARMACOLOGY	-23.0	-2.5	44.7	-8.6	6.3	-2.6	-6.6	-8.6	-2.8	-1.8	-0.491	0.0
214	PSYCHOPHYSIOLOGY	-14.9	8.3	8.8	9.3	8.3	16.9	-13.2	2.9	-6.1	-2.5	-0.466	0.0
215	PURINERGIC SIGNALLING	-11.4	4.9	22.8	0.9	-4.8	5.6	-5.4	-17.8	10.7	33.2	1.396	1.0
216	RESTORATIVE NEUROLOGY AND NEUROSCI-ENCE	-5.9	23.7	1.2	29.3	-12.5	-16.8	-5.1	6.9	-40.4	42.7	-0.548	0.0
217	REVIEWS IN THE NEUROSCIENCES	-12.8	8.0	29.6	55.7	-16.7	1.7	-20.4	-4.0	0.5	1.7	-1.82	0.0
218	SEIZURE-EUROPEAN JOURNAL OF EPILEPSY	-12.1	7.2	26.2	-8.8	-2.6	16.0	16.1	15.8	-11.5	2.7	0.263	1.0
219	SLEEP	-11.3	7.9	21.7	5.1	-11.0	4.3	2.7	4.4	-9.3	-0.7	-0.632	0.0
220	SLEEP AND BIOLOGICAL RHYTHMS	-20.9	17.2	28.2	23.0	14.8	-29.3	47.5	6.8	-22.5	-27.9	-2.538	0.0
221	SLEEP MEDICINE REVIEWS	-7.9	-1.8	20.8	-8.7	-0.8	18.4	22.0	-13.8	-6.9	5.3	0.13	1.0
222	SOCIAL COGNITIVE AND AFFECTIVE NEURO-SCIENCE	-0.8	23.3	-3.8	-2.5	4.6	-11.1	-22.8	-30.8	25.3	16.7	-0.242	0.0
223	SOCIAL NEUROSCIENCE	-16.0	14.3	-7.3	4.4	-16.3	14.2	-18.8	4.4	-7.4	3.2	0.244	1.0
224	SOMATOSENSORY AND MOTOR RESEARCH	-20.1	1.4	8.1	-16.8	4.2	30.4	16.8	21.4	11.1	-38.0	0.606	1.0
225	STEREOTACTIC AND FUNCTIONAL NEUROSUR-GERY	3.5	-12.4	14.7	-14.2	15.6	-2.6	0.1	-16.2	36.7	1.3	1.175	1.0

ID	Journal Name	2022	2021	2020	2019	2018	2017	2016	2015	2014	2013	Slope	Slope sign
226	STRESS-THE INTERNATIONAL JOURNAL ON THE BIOLOGY OF STRESS	-31.1	-4.4	12.6	43.1	-28.8	17.6	8.7	-12.2	-21.6	6.5	0.226	1.0
227	SYNAPSE	-9.3	-1.0	10.5	-8.9	7.8	10.7	5.2	-4.7	-12.4	5.1	0.115	1.0
228	TRANSLATIONAL NEUROSCIENCE	66.1	-28.1	28.0	-32.6	144.7	-9.7	-8.9	-23.3	84.2	48.5	1.747	1.0
229	TRENDS IN COGNITIVE SCIENCES	-18.7	21.0	32.9	-5.9	4.0	1.0	-13.7	-18.7	3.9	32.1	0.319	1.0
230	TRENDS IN NEUROSCIENCES	-6.3	22.7	7.3	4.7	7.6	2.8	-11.0	-7.8	5.1	-5.0	-1.447	0.0
231	VISION RESEARCH	-9.3	5.2	-27.7	19.8	5.3	5.0	11.0	-2.1	-23.8	11.4	0.512	1.0
232	VISUAL NEUROSCIENCE	0.3	-41.5	8.0	82.4	-14.6	10.9	-7.2	-15.2	31.7	13.6	1.657	1.0
233	ZHURNAL VYSSHEI NERVNOI DEYATELNOSTI IMENI I P PAVLOVA	7.9	-36.4	1.2	60.6	-13.5	31.8	34.9	59.1	-48.4	-14.1	-0.147	0.0
